# Multi-species meta-analysis identifies transcriptional signatures associated with cardiac endothelial responses in the ischaemic heart

**DOI:** 10.1093/cvr/cvac151

**Published:** 2022-09-09

**Authors:** Ziwen Li, Emmanouil G Solomonidis, Bronwyn Berkeley, Michelle Nga Huen Tang, Katherine Ross Stewart, Daniel Perez-Vicencio, Ian R McCracken, Ana-Mishel Spiroski, Gillian A Gray, Anna K Barton, Stephanie L Sellers, Paul R Riley, Andrew H Baker, Mairi Brittan

**Affiliations:** Centre for Cardiovascular Science, The Queen’s Medical Research Institute, University of Edinburgh, Edinburgh EH16 4TJ, UK; Centre for Cardiovascular Science, The Queen’s Medical Research Institute, University of Edinburgh, Edinburgh EH16 4TJ, UK; Centre for Cardiovascular Science, The Queen’s Medical Research Institute, University of Edinburgh, Edinburgh EH16 4TJ, UK; Centre for Cardiovascular Science, The Queen’s Medical Research Institute, University of Edinburgh, Edinburgh EH16 4TJ, UK; Centre for Cardiovascular Science, The Queen’s Medical Research Institute, University of Edinburgh, Edinburgh EH16 4TJ, UK; Centre for Cardiovascular Science, The Queen’s Medical Research Institute, University of Edinburgh, Edinburgh EH16 4TJ, UK; Centre for Cardiovascular Science, The Queen’s Medical Research Institute, University of Edinburgh, Edinburgh EH16 4TJ, UK; Centre for Cardiovascular Science, The Queen’s Medical Research Institute, University of Edinburgh, Edinburgh EH16 4TJ, UK; Centre for Cardiovascular Science, The Queen’s Medical Research Institute, University of Edinburgh, Edinburgh EH16 4TJ, UK; Centre for Cardiovascular Science, The Queen’s Medical Research Institute, University of Edinburgh, Edinburgh EH16 4TJ, UK; Division of Cardiology, Centre for Heart Lung Innovation, Providence Research, University of British Columbia, Vancouver, Canada; Department of Physiology, Anatomy and Genetics, University of Oxford, South Parks Road, Oxford OX1 3PT, UK; Centre for Cardiovascular Science, The Queen’s Medical Research Institute, University of Edinburgh, Edinburgh EH16 4TJ, UK; Centre for Cardiovascular Science, The Queen’s Medical Research Institute, University of Edinburgh, Edinburgh EH16 4TJ, UK

**Keywords:** scRNA-seq meta-analysis, ischaemic heart disease, vascular regeneration

## Abstract

**Aim:**

Myocardial infarction remains the leading cause of heart failure. The adult human heart lacks the capacity to undergo endogenous regeneration. New blood vessel growth is integral to regenerative medicine necessitating a comprehensive understanding of the pathways that regulate vascular regeneration. We sought to define the transcriptomic dynamics of coronary endothelial cells following ischaemic injuries in the developing and adult mouse and human heart and to identify new mechanistic insights and targets for cardiovascular regeneration.

**Methods and results:**

We carried out a comprehensive meta-analysis of integrated single-cell RNA-sequencing data of coronary vascular endothelial cells from the developing and adult mouse and human heart spanning healthy and acute and chronic ischaemic cardiac disease. We identified species-conserved gene regulatory pathways aligned to endogenous neovascularization. We annotated injury-associated temporal shifts of the endothelial transcriptome and validated four genes: VEGF-C, KLF4, EGR1, and ZFP36. Moreover, we showed that ZFP36 regulates human coronary endothelial cell proliferation and defined that VEGF-C administration *in vivo* enhances clonal expansion of the cardiac vasculature post-myocardial infarction. Finally, we constructed a coronary endothelial cell meta-atlas, CrescENDO, to empower future in-depth research to target pathways associated with coronary neovascularization.

**Conclusion:**

We present a high-resolution single-cell meta-atlas of healthy and injured coronary endothelial cells in the mouse and human heart, revealing a suite of novel targets with great potential to promote vascular regeneration, and providing a rich resource for therapeutic development.

## Graphical Abstract

**Graphical Abstract:**
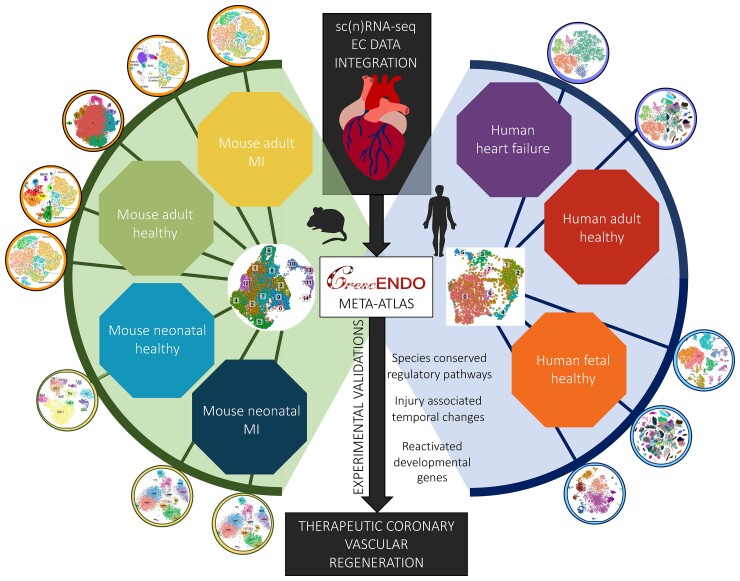
Graphical Abstract outlining the strategy for meta-analysis of integrated single cell and single nucleus RNA-sequencing studies of coronary endothelial cells in mice and humans during developmental and adult stages and in healthy and diseased states. This produced a vast resource of targets with a potential role in coronary vascular regeneration, which can be accessed through the CrescENDO meta-atlas. Specifically, we highlight species-conserved pathways, mapped temporal changes in endothelial cell gene expression in early and late disease stages, and also highlight developmental genes that are reactivated in the injured adult heart. We envisage that this will support future therapeutic strategies to promote coronary neovascularisation following myocardial infarction.

**Time for primary review: 37 days**

## 1. Introduction

The development of heart failure as a complication of myocardial infarction (MI) is termed ischaemic cardiomyopathy (ICM). Despite advances in therapy, it remains a common condition with significant mortality and morbidity, affecting an estimated 64.3 million people worldwide.^1^ Early restoration of blood supply in the infarcted region is critical to the treatment of sequelae of MI, and has been shown to significantly decrease mortality.^2^ Moreover, an impaired function of the microvasculature in the heart has been associated with the development of heart failure.^3^ Therefore, central to regenerative strategies following MI is the requisite for rapid and effective re-establishment of functional blood vascular networks to provide a framework to support cardiomyocyte survival and restore cardiac function.^3^ The mouse and pig heart can extensively, but transiently, regenerate during the first few days of life *via* proliferation and migration of pre-existing cardiomyocytes^4–6^ and also through a robust vasculogenic response with the establishment of new vasculature and penetration of collateral vessels into the infarcted region. Indeed, endothelial cell (EC) migration to the injury site in the neonatal mouse heart precedes cardiomyocyte migration and is critical to provide a vascular infrastructure to support migrating cardiomyocytes as they rebuild muscle.^7^ Long-term functional recovery has been reported in a human newborn heart after MI,^8^ indicating that human neonates may be capable of endogenous cardiac regeneration. Studies by Bergmann and colleagues refuted the long-standing postulation that the adult heart is a post-mitotic organ, showing that adult human cardiomyocytes are capable of renewal throughout life^9^ and, importantly, that coronary vascular ECs have a high turnover rate in the adult human heart of over 15% per year.^10^ However, despite this apparent capacity to retain stable cell numbers in homeostatic conditions, it is clear that intrinsic mechanisms in the adult heart are insufficient to support physiological regeneration following ischaemic injury. The redeployment of developmental signalling systems is a viable paradigm of regenerative medicine, although a better understanding of the underpinning regulatory pathways that must be targeted to facilitate adult myocardial neovascularization is logical.^11^

The rapid implementation of single-cell RNA-sequencing (scRNA-seq) technology has empowered numerous studies interrogating cell state, fate, diversity and function with molecular resolution and in an unbiased fashion, including coronary vessel development and vascular responses following ischaemic injury.^12^ However, the static nature of scRNA-seq analysis can prohibit study of dynamic processes, and clinical relevance is difficult to extrapolate from analysis of non-human tissues. Therefore, we undertook a meta-analysis of integrated scRNA-seq data from studies of developing and adult mouse and human coronary vascular ECs in healthy and injured/diseased states. Meta-analysis normally refers to statistical analyses that identify, appraise, synthesize, and combine the results of independent studies addressing the same scientific questions,^13^ while in our case, it is a specific approach of systematically curating, extracting, integrating, and analysing coronary ECs data from scRNA-seq studies in the healthy and injured mouse and human hearts. We aimed to harmonize molecular signals across these data sets, thereby achieving deeper systemic insight and broadening the scope for biological interpretation and translational opportunity. We constructed a human and mouse coronary EC meta-atlas, CrescENDO, a valuable public resource to foster broad-ranging analysis beyond the scope of this study, for future targeting of pathways associated with coronary EC regeneration and restoration of cardiac function after MI. This meta-analysis provides a powerful approach to map the endothelial transcriptome during development and in response to cardiac injury. Further, we undertook experimental studies to validate a series of targets identified in our study: KLF4, VEGF-C, ZFP36, and EGR1. Finally, we present experimental evidence for a role of ZFP36 in regulation of human cardiac EC proliferation and a therapeutic neovascularization phenotype in the post-ischaemic adult heart induced by administration of VEGF-C in a mouse model of MI.

## 2. Methods

### 2.1 ScRNA-seq meta-analysis workflow

**Figure 1:**
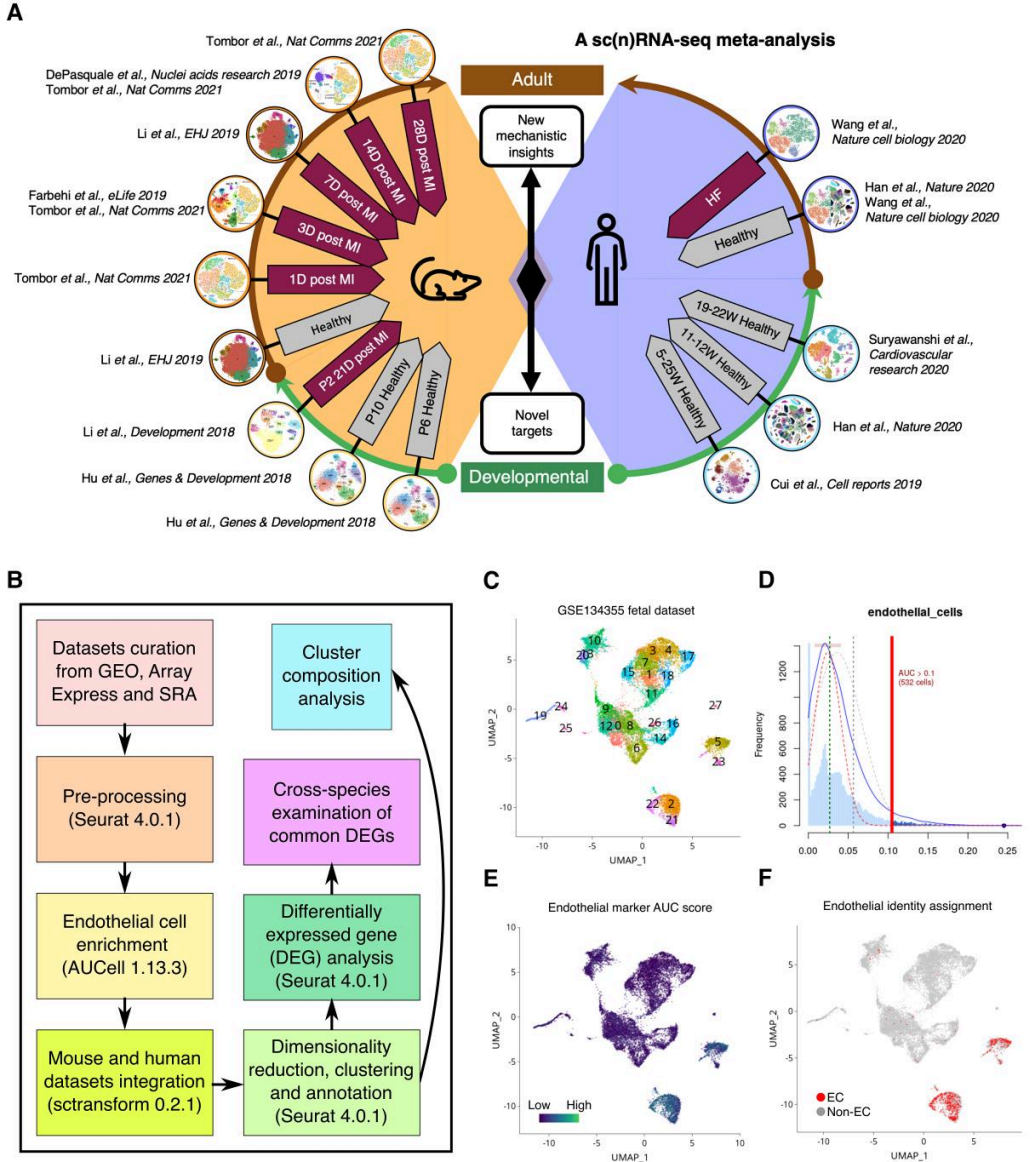
Cross-species meta-analysis of sc(n)RNA-seq data to generate new mechanistic insights and targets for cardiovascular regeneration. (*A*) Schematic overview of the independent sc(n)RNA-seq data sets and original publications of data sets integrated in the meta-analysis. (*B*) Computational workflow of the study. (*C*–*F*) An example of EC enrichment using AUCell package (version 1.13.3). (*C*) The Seurat object generated from GSE134255 was subject to endothelial cell enrichment based on the expression of a panel of 45 endothelial markers. (*D*) The expression of the panel of 45 EC markers in each cell was scored, the proportion of expressed genes and the relative expression values are assessed, and the cut-offs were automatically generated to distinguish cells with high and low EC marker expression based on the distribution of the score. (*E*) All cells were coloured based on the AUC score. (*F*) Cells that passed the calculated threshold were assigned as endothelial cells. Endothelial cells were subset from the original Seurat object to generate a new endothelial-specific Seurat object, containing all the raw counts and metadata for the selected cells.

ScRNA-seq data sets generated from studies of mouse and human cardiac cells were curated from public repositories and pre-processed using the Seurat package^14^ (version 4.0.1; Supplementary material online, *Figure S1A* and *B* and *Table S1*). ECs were extracted from each data set based on the expression of a panel of endothelial markers^15^ (Supplementary material online, *Figure S2A* and *D*) using the AUCell package^16^ (version 1.13.3) to form endothelial-specific data sets (*Figure 1C–F*). Mouse and human data sets were integrated respectively using sctransform^17^ (version 0.2.1) after low-quality cells were removed, and two data sets (Seurat objects) were generated. Dimensionality reduction, unsupervised clustering (i.e. where EC with similar gene expression profiles were grouped together to visualize heterogeneity using Seurat) and gene expression analysis were then performed on the new data sets. Markers of each cluster were identified, and their putative functions were predicted. Cluster composition was analysed for each group (defined by age, condition, and disease stage). Differentially expressed genes (DEGs) were identified between different groups within each species. The following comparisons were performed for the mouse data: (i) DEG analysis of injured vs. uninjured ECs from mouse hearts within (<P7) and outside (>P7) of the regenerative window to identify the similarity and differences in gene expression in response to ischaemic injury, (ii) DEG analysis of ECs from injured adult mouse hearts at multiple timepoints post-MI vs. ECs from the uninjured adult mouse heart to identify temporal changes in gene expression. The following comparisons were performed for the human data: (i) DEG analyses between uninjured adult ECs vs. patients with cHF (heart failure caused by ICM) and uninjured adult ECs vs. patients with dHF (heart failure caused by dilated cardiomyopathy) to reveal similarities and differences in the endothelial transcriptome in heart failure patients of different aetiology, (ii) DEG analysis between uninjured foetal and uninjured adult ECs and comparisons of DEGs in (i) and (ii) to identify up- and down-regulated genes shared by both uninjured foetal and injured adult ECs compared with the uninjured adult ECs. DEGs identified between uninjured vs. injured ECs in both mouse and human were cross-examined to reveal injury-induced changes in gene expression common between the two species.

#### 2.1.1 Data set selection

Data sets and corresponding metadata (repository, accession number, species, age, health condition, and cell source) from single-cell RNA-sequencing studies of mouse and human cardiac cells published between 2018 and 2021 were curated from public transcriptomics data repositories Gene Expression Omnibus (GEO),^18^ ArrayExpress,^19^ and Sequence Read Archive (SRA) database^20^ (Supplementary material online, *Figure S1* and *Table S1*). The mouse data sets used in this study included: GSE117893 (P2 21 days post-MI), GSE118545 (P6 and P10 healthy), E-MTAB-7476 (adult 3 days post-MI), GSE132880 (adult healthy and 7 days post-MI), GSE136088 (adult 14 days post-MI), and E-MTAB-9816 (adult 1, 3, 14, and 28 days post-MI). The human included: GSE106118 (foetal healthy), GSE134355 (foetal and adult healthy), SRP234812 (foetal healthy), GSE109816 (adult healthy), and GSE121893 (adult cHF and dHF).

#### 2.1.2 Data set pre-processing

The finalized raw gene expression data from GEO and ArrayExpress was loaded into R (version 4.0.4) and pre-processed using Seurat^14^ (version 4.0.1). Cells with low (≤200) or abnormally high (≥4000) gene counts and/or a high percentage of mitochondrial genes (≥20%) were removed, and data normalized using sctransform^17^ (version 0.2.1), which dampens the influence of technical characteristics (such as sequencing depth), while preserving biological variation. SCTranformed biological replicates were subsequently integrated to generate one single Seurat object: first, highly variable features were selected for integration using **SelectIntegrationFeatures** function (*nfeatures* = 3000); second, the selected features were used to prepare individual objects using **PrepSCTIntegration** function; third, the integration anchors were decided using **FindIntegrationAnchors** (*normalization.method* = *‘SCT’, anchor.features* = *features*); finally, the data sets were integrated using **IntegrateData** function (*normalization.method* = *‘SCT’*). Downstream dimensionality reduction was carried out using principal component analysis (PCA) by running function **RunPCA** on integrated data with default settings from Seurat and clusters visualized using uniform manifold approximation and projection (UMAP) by running function **RunUMAP** with default settings from Seurat. A **set.seed(1024)** step was run before the **RunUMAP** step to ensure the reproducibility of UMAP projections. The updated data set was then subject to **FindNeighbors** function (*dims* = 1:30). The resolution parameter used for finding clusters was determined by iterating all resolutions in the range of 0.1 to 2 with a 0.1 step using **FindClusters**, generating the corresponding clustering result, and visualizing the relationships among the results using clustree^21^ (version 0.4.3): the final resolution was chosen when the clustering began to stabilize.

#### 2.1.3 EC enrichment

In order to include only EC data in our integrated data sets and to remove non-EC transcriptomic information, i.e. from data sets where the original study was of whole heart preparations, Seurat objects were subject to EC enrichment based on the expression of a panel of 45 endothelial markers^15^ (Supplementary material online, *Figure S2A* and *D*). All cells were scored for the expression of the endothelial markers and ranked using the AUCell package^16^ (version 1.13.3; *Figure 1C–F*). AUCell took the 45 EC markers as input and output the gene set ‘activity’ in each cell. It calculated the enrichment of these markers as an area under the recovery curve (AUC) across the ranking of all genes in each cell, where genes were ranked by their expression value. The scoring method was based on a recovery analysis, where the *x*-axis was the ranking of all genes based on expression level; the *y*-axis was the number of genes recovered from the input set. AUCell then used the AUC to calculate whether the set of the 45 EC markers was enriched at the top of the ranking for each cell. In this way, the AUC represented the proportion of expressed genes in the EC signature and their relative expression values compared with the other genes within the cell. The output is a matrix with the AUC scores for each cell. Then, we generated a binary matrix using a cut-off of the AUC sore for the EC marker set to distinguish ECs and non-ECs. These cut-offs were determined automatically, or manually adjusted by inspecting the distribution of the AUC scores. Cells that passed the calculated threshold were assigned as ECs, and the expression of *Pecam1* (mouse)/*PECAM1* (human), the CD31 protein coding gene, was plotted to confirm endothelial identity (*Figure 1C–F*). ECs were subset from the original Seurat object to generate a new endothelial-specific Seurat object, containing all the raw counts and metadata for the selected cells (*Table 1*).

**Table 1:** Numbers of endothelial cells in each group in the meta-analysis following pre-processing and endothelial cell enrichment

Group	Number of cells
Mouse P6 uninjured	1510
Mouse P10 uninjured	1211
Mouse P2 21 days post-MI	270
Mouse adult uninjured	2747
Mouse adult 1 day post-MI	59
Mouse adult 3 days post-MI	635
Mouse adult 7 days post-MI	5168
Mouse adult 14 days post-MI	454
Mouse adult 28 days post-MI	177
Human foetal uninjured	1304
Human adult uninjured	551
Human adult cHF	132
Human adult dHF	264

P, postnatal day; MI, myocardial infarction; cHF, heart failure caused by ischaemic cardiomyopathy; dHF, heart failure caused by dilated cardiomyopathy.

#### 2.1.4 Data set integration

Genes with a subcellular localization in mitochondria downloaded from the Mouse.MitoCarta2.0 and Human.MitoCarta2.0^22^ as well as those with typical mitochondrial gene names (beginning with ‘mt’ and ‘MT’) were removed from individual enriched endothelial-specific data sets and excluded from the downstream analyses,^23^ since increased numbers of mitochondrial genes are likely associated with poor data quality.^24^ The resulting data sets were normalized and integrated using the sctransform package into new data sets. A second round of dimensionality reduction, clustering, and cluster annotation was performed as in the pre-processing step (*dims* = 1:50). Marker genes for each cluster were defined as the DEGs between the cluster and the rest of the cells, with a minimum log_2_-fold change in average expression of 0.25 using a Wilcoxon rank sum test. Clusters with high expression of the inflammatory cell and haematopoietic marker *Ptprc* (CD45) were identified and removed where applicable. A third round of dimensionality reduction, clustering, and cluster annotation was then performed on the new ‘clean’ data set. Marker genes in each cluster were then defined using the aforementioned method and ranked using the adjusted *P-*values and the average log_2_-fold change. Putative functions for each cluster were predicted using GeneMANIA^25^ and topGO^26^ (version 2.42.0). Finally, we integrated all data sets and performed dimensional reduction and clustering.

#### 2.1.5 Cluster composition analysis

The number of cells in each cluster from each group (defined by the species, age, health condition, disease state) and the percentage of cells comprising each cluster within the group were calculated, and the percentage data normalized across all groups. This revealed the change in cluster composition between groups, especially the shift of majority clusters based on each different grouping factor.

#### 2.1.6 DEG analysis

For DEG analysis, **DefaultAssay** function from Seurat was used to set the current assay to ‘RNA’, the counts were normalized using **NormalizeData** function (*normalization.method* = *‘LogNormalize’, scale.factor* = 10 000), and the normalized counts were used for DEG analysis. DEG analyses were carried out between different groups: first, an **Idents** function was run to switch to the category (e.g. age, injury/disease status) where comparisons needed (e.g. foetal vs. adult), then up- and down-regulated genes were identified using **FindMarkers** function with a minimum log_2_-fold change in average expression of 0.25 using a Wilcoxon rank sum test (adjusted *P* < 0.05). These genes were then cross-examined to identify those that were common to multiple groups and those that were specifically differentially expressed in a single group, with results depicted in Venn diagrams. Average expression and percentage of cells expressing the DEGs were visualized using **DotPlot** function using the ‘RNA’ assay and ‘data’ slot.

#### 2.1.7 Code availability

Codes used to generate results in this study are available on GitHub. A shiny app CrescENDO (www.crescendo.science) was developed to allow further exploration of the data included in the study.

#### 2.1.8 CrescENDO user instruction

Choose the species and available data sets from the sidebar on the ‘Home’ tab and launch the app by clicking the ‘Launch’ button. A selection box will appear on the main panel called ‘View multiple genes of interest’. This allows visualization of the expression of multiple genes of choice within specific data sets in the format of dot plots. The size of the dot represents the percentage of cells expressing the gene(s) of interest and the colour indicates the average expression level. ‘Reset’ button resets all inputs from you to allow new inputs to be entered.

We request that users of CrescENDO acknowledge/cite the current manuscript in any related publications.

### 2.2 The role of VEGF-C in activation of endogenous neovascularization by coronary EC in the adult mouse heart post-MI

#### 2.2.1 rhVEGF-C treatment in *Pdgfb-iCreER^T2^-R26R-Brainbow2.1* mice following ischaemic injury induced *via* permanent coronary artery ligation

Experiments were performed in accordance with the Guide for the Care and Use of Laboratory Animals prepared by the Institute of Laboratory Animal Resources and approved by the UK Home Office and the University of Edinburgh Animal Welfare and Ethical Review Committee. Male and female mice (aged 12 weeks) that were heterozygous for both the *Brainbow2.1* and *Pdgfb-iCreER^T2^* transgenes were used.^27^ A single dose of tamoxifen (150 mg/kg in 200 μL peanut oil) was administrated *via* intraperitoneal injection to induce Cre-recombination and subsequent expression of one of four fluorescent proteins, mCFP, YFP, nGFP, or RFP from the *Brainbow2.1* transgene specifically in *Pdgfb* lineage ECs. Fluorophore expression is inherited by daughter cells following cell division and therefore this model allowed us to directly visualize and quantify blood vessel network expansion *via* EC clonal proliferation in response to MI with/without rhVEGF-C treatment. At 14 days post-tamoxifen, the mice underwent surgically induced MI by permanent ligation of the left anterior descending coronary artery.^27^ Mice were anaesthetized using ketamine/xylazine (intraperitoneal, 100 and 10 mg/kg, respectively), intubated and ventilated at a weight-appropriate volume and frequency. A left thoracotomy was performed, the pericardium was opened, and a surgical suture was placed around the proximal left anterior descending coronary artery to induce MI. After thorax closure and air removed from the chest cavity, atipamezole (antisedan) reversal (intraperitoneal, 1.0 mg/kg) was given subcutaneously. Intubation was maintained until the animal regained the ability to breathe spontaneously. Homeothermic support was provided until the animal was able to independently regulate body temperature. The animals were allowed to recover with aseptic precautions and received post-operative buprenorphine analgesic (subcutaneous, 0.05 mg/kg) during recovery. Mice were given recombinant human VEGF-C (0.1 μg/g in 100 μL, *n* = 4) or phosphate-buffered saline (PBS; as a vehicle control, 100 μL, *n* = 2) intraperitoneally just after recovery and at 2, 4, and 6 days post-surgery.

#### 2.2.2 Tissue collection and processing

At 7 days post-surgery, mice were euthanized with pentobarbitone (intraperitoneal, 40 mg in 200 μL volume) and perfusion fixed by infusion of PBS followed by 4% (w/v) paraformaldehyde *via* left ventricle. Hearts were dissected and placed in 4% paraformaldehyde overnight at 4°C. Wholemount sections were prepared where hearts were embedded in 4% agarose (Sigma-Aldrich, Darmstadt, Germany) and sectioned at 100 μm using a Compresstome® VF-300 Vibrating Microtome (Precisionary Instruments, Livingston, UK). Wholemount sections were stored in PBS with 0.025% sodium azide (Sigma-Aldrich) at 4°C. Some wholemount sections were further embedded in OCT, re-sectioned at 10 μm using a cryostat (Thermo Fisher Scientific, Oxford, UK), and stored at −80°C.

#### 2.2.3 Immunofluorescence

Serial cryosections for immunohistochemical analyses were permeabilized in 0.5% Tween-20 in PBS (PBS-T) for 30 min and blocked in 10% normal goat serum (NGS; Thermo Fisher Scientific) plus 1% BSA in PBS-T for 1 h at room temperature. Primary antibodies (goat IgG anti-Flt4 at 1:50; R&D System AF743, Minneapolis, MN, USA; rabbit IgG anti-Nrp2 at 1:500, Invitrogen PA5-77526, Inchinnan, UK) were diluted in the blocking solution and cryosections were incubated at 4°C overnight. After further washes in PBS-T (2 × 15 min), sections were incubated with fluorescence-conjugated secondary antibodies (goat anti-rabbit Alexa Fluor 647 at 1:400, A21245 Invitrogen) diluted in the blocking solution for 1 h at room temperature and washed in PBS. Cryosections were mounted in Fluoromount-G™ with 4′,6-diamidino-2-phenylindole (DAPI) (Invitrogen).

#### 2.2.4 Confocal imaging and quantification

Wholemount sections were mounted in RapiClear 1.47 (SunJin Lab, Seoul, South Korea) and imaged using a Zeiss LSM780 confocal microscope with laser lines and detectors as follows: DAPI (405 and 417–508 nm), CFP (458 and 454–502 nm), GFP (488 and 498–506 nm), YFP (514 and 525–560 nm), and RFP (561 and 565–650 nm). Image stacks were acquired at the infarct border regions using 20× Plan Apo VC/NA 0.8 objective at 2 μm Z-step with a total thickness of 50–60 μm. Images were processed and analysed using Fiji v2.0 (ImageJ, Derby, UK) and the clone volume was quantified using Imaris v9.0 (Bitplane, Abingdon, UK). For Flt4 and Nrp2 immunofluorescence, thin sections were imaged using the following laser lines and detectors: DAPI (405 and 417–508 nm) and AlexaFluor 647 (633 and 641–758 nm). Three 850.19 μm × 850.19 μm regions of interests (ROIs) were acquired using 20× Plan Apo VC/NA 0.8 objective at 3 μm Z-step with a total thickness of 12 μm, for each of the healthy (randomly) and ischaemic heart (focused on infarct border region) tissue sections (*n* = 5 for each group), with complete tissue coverage. The total number of Flt4^+^ and the Nrp2^+^ vessels were quantified within the defined ROI, respectively.

### 2.3 Protein level validation of KLF4, EGR1, and ZFP36 expression in cardiac tissues from patients with ICM

#### 2.3.1 Human cardiac samples

Cardiac tissue samples were obtained from patients in Supplementary material online, *Table S3*. The mean age of control subjects was 44 (range 25–63 years, *N* = 5, 100% male). The mean age of patients with ischaemic heart disease was 55.8 (range 45–70, *N* = 5, 60% male).

Cryosections (10 μm) were stained using Masson’s trichrome (Abcam, Cambridge, UK) and haematoxylin and eosin (H&E). Serial cryosections for immunohistochemical analyses were permeabilized in 0.5% Tween-20 in PBS (PBS-T) for 30 min and blocked in 10% NGS (Thermo Fisher Scientific) plus 1% BSA in PBS-T for 1 h at room temperature. Primary antibodies (rabbit IgG anti-human KLF4 at 1:200; Cell Signalling #4038; mouse IgG1 anti-human/mouse ZFP36 at 1:25, Invitrogen # MA5-24972; rabbit IgG anti-EGR1 at 1:200, Cell Signalling #4154; mouse IgG1 anti-human CD31 at 1:100, abcam ab24590; rabbit IgG anti-human CD31 at 1:100, abcam ab23864) were diluted in the blocking solution and cryosections were incubated at 4°C overnight. After further washes in PBS-T (2 × 15 min), sections were incubated with fluorescence-conjugated secondary antibodies (goat anti-mouse Alexa Fluor 488 at 5 μg/mL, A11001 Invitrogen; goat anti-rabbit Alexa Fluor 647 at 5 μg/ml, A21245 Invitrogen; goat anti-mouse Alexa Fluor 568 at 5 μg/mL, A11004 Invitrogen) diluted in the blocking solution for 1 h at room temperature and washed in PBS. Cryosections were mounted in Fluoromount-G™ with DAPI (Invitrogen). Sections were imaged using a Zeiss LSM780 confocal microscope with laser lines and detectors as follows: DAPI (405 and 417–508 nm), Alexa Fluor 488 (488 and 498–579 nm), Alexa Fluor 568 (568 and 570–695 nm), and Alexa Fluor 647 (633 and 641–744 nm). Three 708.49 μm × 708.49 μm ROIs were chosen for each of the healthy and ischaemic heart tissue sections (*n* = 5 for each group). Cell counting for CD31^+^ cells, EGR1^+^ cells, CD31^+^ KLF4^+^ cells, CD31^+^ EGR1^+^ cells was performed using the Cell Counter plug-in in Fiji v2.0 (ImageJ).

### 2.4 *ZFP36* siRNA gene silencing and EC proliferation assay

#### 2.4.1 Cell transfections

Human Cardiac Microvascular ECs (HCMECs, C-12285; PromoCell, Heidelberg, Germany) were maintained in collagen-coated cell culture flasks (CORNING). Three individual lines at Passage 4–5 HCMECs were seeded in collagen-coated six-well plates (Biocoat Collagen I Cell ware, CORNING 356400) in triplicates at 3–5 × 10^4^ cells per well in EC Basal Medium MV2 (C-22221; PromoCell) supplemented with EC Growth Medium MV2 SupplementPack (C-39221; PromoCell). Five nanomolars of control (Silencer™ Select Negative Control No. 1 siRNA 4390843 and Silencer™ Select Negative Control No. 2 siRNA 4390846; Thermo Fisher Scientific) or *ZFP36* siRNA oligonucleotides (Silencer™ Select ZFP36 s14977, s14978, and s14979; Thermo Fisher Scientific, Supplementary material online, *Table S3*) were transiently transfected into HCMECs using Lipofectamine™ RNAiMAX transfection reagent (13778030; Thermo Fisher Scientific) for 6 h and further incubated for 48 h before RNA extraction.

#### 2.4.2 RNA isolation and qrt-polymerase chain reaction

Total RNA was extracted from HCMECs 48 h after transfection using the Qiagen RNeasy Mini Kit (Qiagen, Manchester, UK), according to manufacturer’s instructions. cDNA was synthesized from 100 ng of total RNA using TaqMan™ Reverse Transcription Reagents (Thermo Fisher Scientific). Individual 10 µL Taqman real-time polymerase chain reaction (PCR) reactions consisted of 1.5 µL of cDNA, 5 µL of 2× Taqman mastermix and 0.5 µL of FAM-labelled *ZFP36* probe (Hs00185658_m1; Thermo Fisher Scientific) in 3 µL RNase-free water. The PCR was carried out on a QuantStudio 5 Real-Time PCR system using the following cycling conditions: 10 min at 95°C and 40 cycles of 15 s at 95°C, 60 s at 60°C. All experiments included three no-template controls and were carried out with three biological replicates (one for each HCMEC line) and three technical replicates for all treatment groups, including ‘Cells only control’, ‘Vehicle control’, ‘Control siRNA’, and ‘*ZFP36* siRNA’ groups. For normalization of quantification, housekeeping gene *UBC* (Hs01871556_s1; Thermo Fisher Scientific) was amplified simultaneously. The ΔCt values were calculated as the differences between the Ct values of *ZFP36* and *UBC* and the mean of the ΔCt values from the ‘Cells only control’ groups was subsequently used to calculate the ΔΔCt values and RQ (2^^−ΔΔ*Ct*^) values. The gene expression level was presented in the graph using the RQ values, whereas the statistical analyses were using one-way analysis of variance (ANOVA) and Dunnett’s multiple comparisons tests.

#### 2.4.3 Cell proliferation assay

HCMECs were seeded onto collagen-coated coverslips in 24-well plates (Collagen, Type I solution from rat tail, SIGMA-ALDRICH C3867-1VL) in EC Basal Medium MV2 (C-22221; PromoCell) supplemented with EC Growth Medium MV2 SupplementPack (C-39221; PromoCell). Cells were incubated with 0.2% foetal bovine serum (HyClone, Cramlington, UK) for 2 h after the initial siRNA knockdown and then treated with media containing 10 µM EdU (5-ethynyl-2′-deoxyuridine, supplied in Click-iT™ EdU Alexa Fluor™ 647 Imaging Kit, Thermo Fisher Scientific C10340 and reconstituted in DMSO) and 10% foetal bovine serum (HyClone) for 6 h. Coverslips were then fixed in 4% paraformaldehyde in PBS for 15 min at room temperature, permeabilized using 0.5% Triton X-100 in PBS for 20 min at room temperature and incubated in Click-iT™ reaction cocktail (prepared as per manufacturer’s instructions) for 30 min at room temperature, protected from light. The coverslips were then washed in 3% BSA (KPL 10% BSA Diluent/Blocking Solution Kit; Seracare, Milford, MA, USA) in PBS twice and mounted on slides with Fluoromount-G™ Mounting Medium with DAPI (Thermo Fisher Scientific).

#### 2.4.4 Image acquisition and analysis for cell proliferation assay

A Zeiss LSM 780 confocal microscope equipped with 20× Plan-Apochromat 20×/0.8 M27 objective was used for image acquisition with laser lines and detectors as follows: DAPI (405 and 417–508 nm), Alexa Fluor 488 (488 and 498–579 nm), and Alexa Fluor 647 (633 and 641–744 nm). Three 708.49 μm × 708.49 μm ROIs were chosen for each of the seven treatment groups for each cell line studied. Cell counting for DAPI^+^ and DAPI^+^ EdU^+^ cells was performed using the Cell Counter plug-in in Fiji v2.0 (ImageJ).

### 2.5 Statistical analyses

Statistical analyses were conducted using GraphPad Prism version 9.1.0. Results are expressed as mean ± SD and data were analysed using parametric unpaired *t*-test or one-way ANOVA.

## 3. Results

### 3.1 Cross-species meta-analysis of sc(n)RNA-seq data to generate new mechanistic insights and targets for cardiovascular regeneration

Recent sc(n)RNA-seq technologies have significantly advanced our understanding of the heterogenous cell populations and their dynamic transcriptional profiles in the healthy and diseased heart.^27–36^ However, these stand-alone studies tend to lack full statistical power and typically provide only a ‘snap-shot’ of complex diseases in a single species. Meta-analyses combine the results of independent studies addressing the same scientific question and derive a pooled estimate,^13^ thus can be readily adapted to study sc(n)RNA-seq data from multiple sources to generate robust insights. In this study, we took a meta-analysis approach (*Figure 1A*) to study coronary ECs from developing and adult mouse and human hearts spanning healthy and diseased states. We systematically curated 18 data sets from 11 independent sc(n)RNA-seq studies published between 2018 and 2021 (*Figure 1A*, Supplementary material online, *Figure S1A*), performed EC enrichment for each data set (*Figure 1C–F*), integrated the extracted data for each species, carried out dimensionality reduction, clustering, cluster composition, and DEG analyses, and examined conserved DEGs between mouse and human (*Figure 1B*).

### 3.2 Analysis of neonatal and adult mouse coronary vascular EC data sets reveals 15 transcriptionally distinct cell clusters

**Figure 2:**
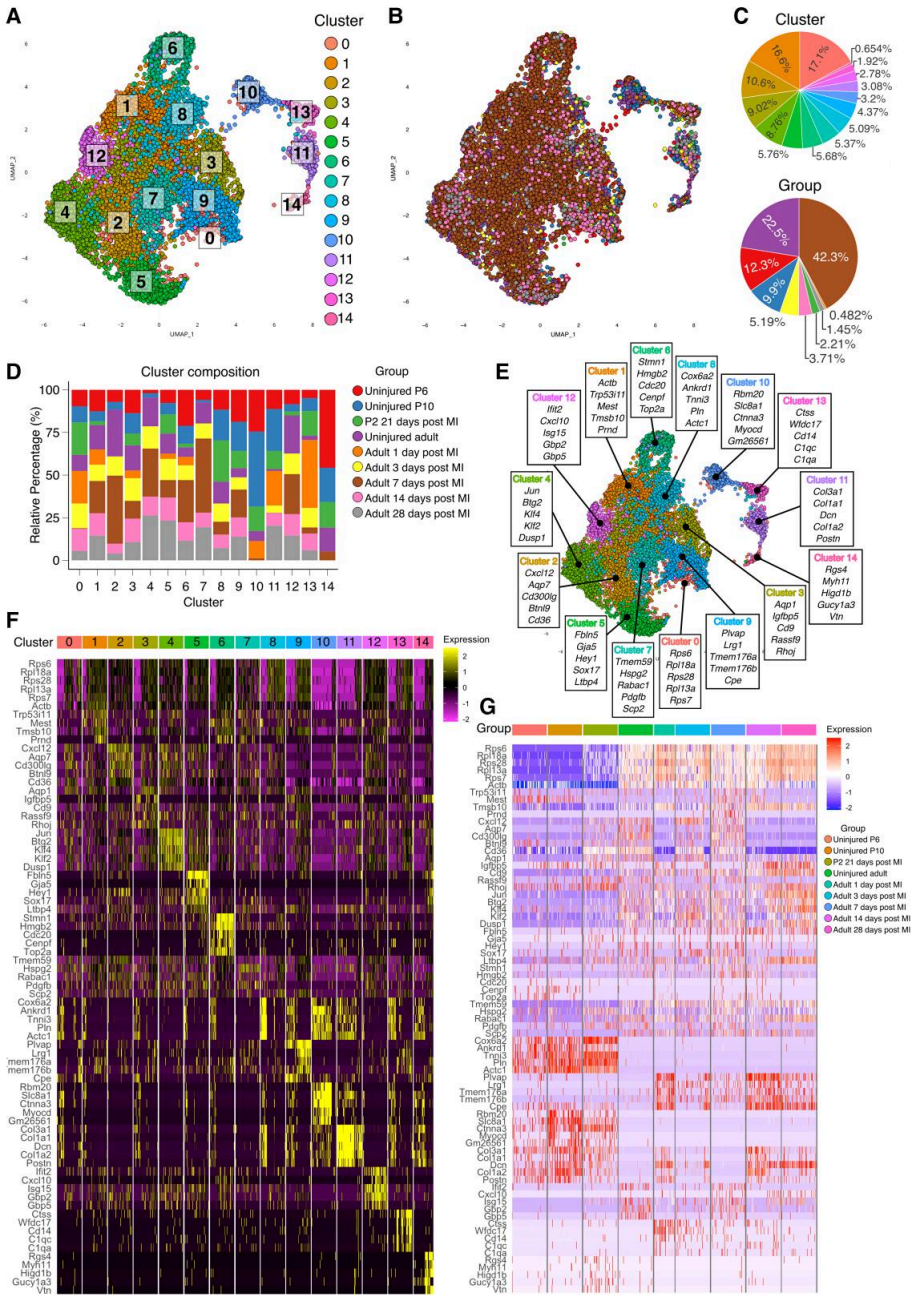
A single-cell transcriptomic map of mouse coronary endothelial cells. (*A*) UMAP plot of 12 231 mouse coronary ECs extracted from public transcriptomic data sets where each spot represents a single cell. Unsupervised clustering analysis identified 15 heterogenous EC clusters in uninjured and post-ischaemic neonatal and adult mouse hearts. Cells are colour coded for each associated EC state. (*B*) Cells in the UMAP plot are colour coded for each group. (*C*) Top, the percentage of ECs from each cluster; bottom, the percentage of ECs from each group. (*D*) Relative percentage of ECs from each cluster across all groups to resolve the cluster composition. (*E*) Top expressed genes for each cluster were identified via DEG analysis, comparing each cluster against the remaining ECs. The top five expressed genes for each cluster are provided. (*F*) Heatmap showing the expression of top cluster markers listed in *E* for each cluster. (*G*) Heatmap showing the expression of the top markers for each group.

Single-cell RNA-sequencing (scRNA-seq) data sets generated from studies of mouse cardiac cells were curated from public repositories and pre-processed using the Seurat package^14^ (version 4.0.1; Supplementary material online, *Figure S1A* and *Table S1*). ECs were extracted from each data set based on the expression of a panel of endothelial markers^15^ (Supplementary material online, *Figure S2A*) using the AUCell package^16^ (version 1.13.3) to form endothelial-specific data sets. Unsupervised clustering was undertaken on 12 231 mouse coronary vascular ECs (*Figure 2A*) extracted across nine different groups spanning neonatal (regenerative) and adult (non-regenerative) stages in healthy states and at early and late timepoints post-MI (Supplementary material online, *Figure S1A* and *Table S1*). This revealed 15 distinct endothelial clusters or ‘states’ (*Figure 2A–C*). Cells from all groups were distributed within each of the clusters, thereby confirming successful data integration across independent studies (*Figure 2D* and *Table 2*). GO-term analysis was applied to reveal putative common functions for each cluster (Supplementary material online, *Table S5*). Analysis of DEGs was carried out to identify the top genes enriched within cells in each cluster (*Figure 2E* and *F*). The expression of the top cluster markers was also examined in each group (*Figure 2G*).

**Table 2:** Mouse coronary endothelial cell cluster composition analysis

	Neonatal coronary ECs (%)			Adult coronary ECs (%)					
Cluster	Regenerative timepoint (<P7)	Non-regenerative timepoint (>P7)		Uninjured	Days post-MI				
	Uninjured	Injured	Uninjured		1	3	7	14	28
0	9.7	19.1	9.4	9.4	19.1	14.5	0.3	13.0	5.5
1	12.6	2.3	5.8	14.3	8.9	9.6	19.0	13.2	14.4
2	7.1	0	4.5	27.5	0	11.2	39.9	5.8	4.0
3	13.7	7.0	12.0	14.7	4.5	13.3	10.4	13.9	10.6
4	2.0	0	2.5	16.9	0	13.0	28.2	11.1	26.2
5	7.7	11.3	6.7	11.8	3.0	12.4	10.3	13.4	23.3
6	21.1	2.1	10.5	5.6	4.9	8.7	24.8	10.8	11.5
7	6.0	0	1.4	12.4	0	8.8	43.5	8.5	19.4
8	11.6	24.3	18	12.8	0	10.7	7.5	7.9	7.2
9	18.8	10.1	17.1	5.3	0	7.2	16.3	11.4	13.8
10	24.5	14.4	43.9	5.9	10.1	0	1.2	0	0
11	11.3	10.7	24.5	1.1	20.2	4.3	0.1	7.5	20.2
12	8.5	2.4	4.2	22.3	0	12.1	23.5	12.7	14.4
13	4.8	14.5	7.5	2.6	39.8	11.5	3.6	9.8	5.9
14	45.7	15	20.1	14.0	0	0	5.1	0	0

The percentage (%) of ECs in each cluster was calculated for each group and normalized across all groups.

P, postnatal day; ECs, endothelial cells; MI, myocardial infarction.

### 3.3 Common and distinct gene expression programmes are activated in neonatal and adult mouse coronary ECs following MI

**Figure 3:**
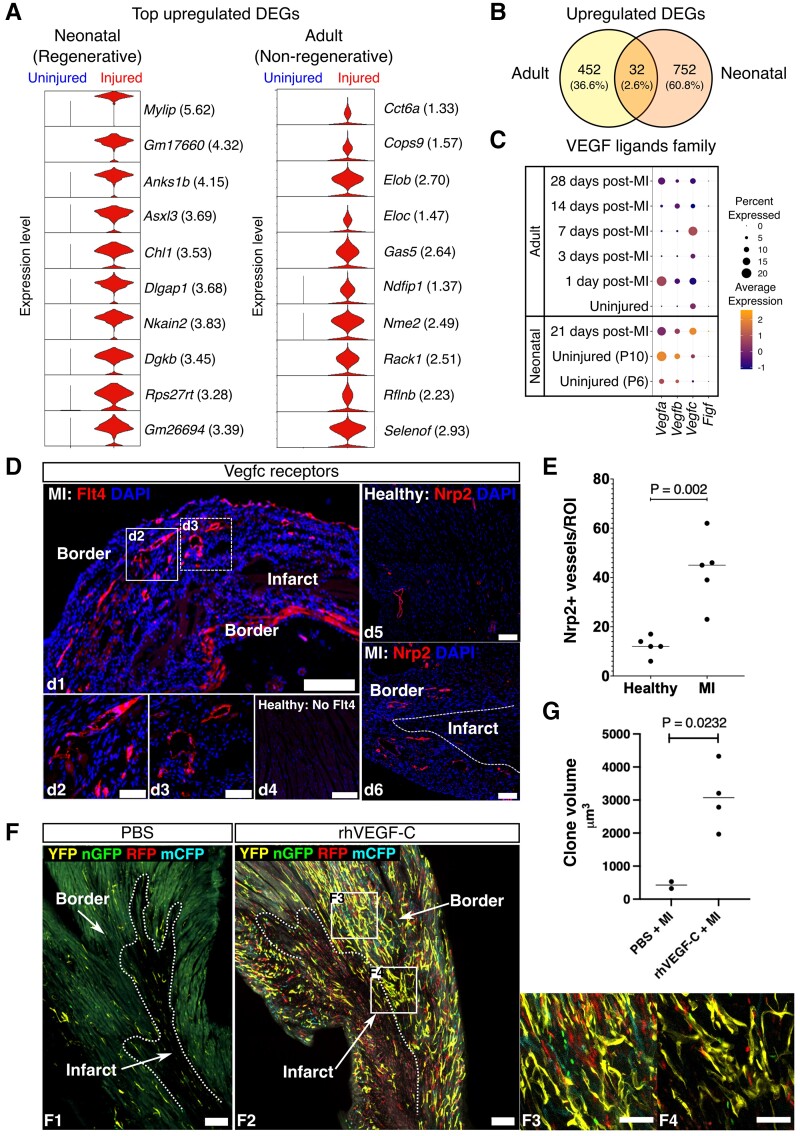
Redeployment of developmental mechanisms governing cardiac regeneration promotes neovascularization in adult heart. (*A*) Left, top up-regulated DEGs (log_2_FC) in injured vs. uninjured coronary ECs in neonatal hearts within the 7-day window of regeneration; right, top up-regulated DEGs (log_2_FC) in injured vs. uninjured coronary ECs in adult hearts. (*B*) Numbers and percentages of up-regulated DEGs common to both adult and neonatal coronary ECs (32, 2.6%) and those specific to each age group (adult: 452, 36.6%; neonate: 752, 60.8%). (*C*) Expression levels (indicated by colour gradient) of the VEGF family ligands, *Vegfa*, *Vegfb*, *Vegfc*, *Vegfd/Figf*, and the percentage of cells expressing these genes in different subgroups of neonatal and adult mouse coronary ECs. (*D*) Representative immunostaining of Flt4 and Nrp2 (VEGF-C receptors) expression in the left ventricles of adult mouse hearts at 7 days post-MI compared with healthy adult mouse hearts. Left, Flt4 showed high levels of expression in the infarct border region of the injured heart (d1) but was absent from the healthy heart (d4). High-power images (d2, d3) show Flt4^+^ vasculature in the white-boxed regions of the lower power images. Right, the number of Nrp2^+^ vessels was increased in the injured heart (d5), compared with the healthy heart (d6). Scale bars: 200 μm (d1), 50 μm (d2, d3), and 100 μm (d4, d5, and d6). (*E*) Nrp2^+^ vessels were quantified in the infarct border region of the mouse heart at 7 days post-MI compared with the healthy mouse heart (Nrp2^+^ vessel number per region = 43.0 ± 14.1 vs. 12.2 ± 4.1, *P* = 0.002, unpaired *t*-test). (*F*) Representative images of wholemounts of the injured left ventricle from PBS-treated (f1) and rhVEGF-C-treated (f2) adult mouse hearts showing a striking increase in EC-specific fluorophore expression following rhVEGF-C administration. High-power images (f3 and f4) show large vascular fluorescent clones in the white-boxed areas in the infarct border region of the rhVEGF-C-treated hearts post-MI. Scale bars: 100 μm (f1, f2), 50 μm (f3, f4). (*G*) Quantification of the volume of vascular EC clones expressing Brainbow2.1 fluorophores (YFP, nGFP, RFP, or mCFP) in coronary ECs showed increased neovascularization in the infarct border in rhVEGF-C-treated hearts compared with the PBS controls [clone volume (μm^3^) = 3072 ± 491.2 vs. 426 ± 105, *P* = 0.02; unpaired *t*-test]. A clone was defined as two or more adjacent ECs expressing the same fluorescent protein.

We identified the top up-regulated DEGs in ischaemic vs. healthy mouse coronary ECs within the 7-day window of regeneration in the neonatal mouse heart.^4,5^*Mylip*, *Gm17660*, *Anks1b*, *Asxl3*, and *Chl1* were among the top identified up-regulated genes (*Figure 3A*). We then identified the top up-regulated DEGs in injured vs. uninjured coronary ECs specifically from the adult mouse data sets only, where cells from all timepoints post-MI (Days 1, 3, 7, 14, and 28) were grouped together in the first instance. This showed an enrichment of genes such as *Cct6a*, *Cops9*, *Eloc*, *Ndfip1*, *Rack1*, and selenium response-related genes in injured adult ECs after MI (*Figure 3A*). Interestingly, these modular analyses of gene expression in injured vs. uninjured coronary ECs in both the neonatal and adult groups identified 32 (2.6%) genes that were common to both comparisons (*Figure 3B* and *Table 3*). This may signify developmental and/or regenerative gene expression programmes in the neonatal mouse heart vasculature that are reactivated in adult coronary ECs post-MI during endogenous attempts at neovascularization.

**Table 3:** Top up-regulated DEGs in both neonatal and adult mouse heart endothelial cells after MI

Gene name	Neonatal mouse coronary ECs post-MI		Adult mouse coronary ECs post-MI	
	Rank	Log_2_FC	Rank	log_2_FC
*Sox18*	398/785	0.71	85/485	1.01
*Rbpms*	376/785	0.77	115/485	0.73
*Hspa8*	353/785	0.89	128/485	0.63
*Nedd9*	667/785	0.28	195/485	0.57
*Rps28*	396/785	0.75	134/485	0.52
*Cdh13*	449/785	0.7	197/485	0.49
*Rpl3*	762/785	0.43	120/485	0.49
*Slc25a4*	784/785	0.31	228/485	0.42
*Adam15*	609/785	0.52	434/485	0.39
*Wwc2*	498/785	0.53	313/485	0.38
*Tsc22d3*	364/785	0.53	392/485	0.37
*H2afj*	686/785	0.4	239/485	0.36
*Nptn*	622/785	0.36	283/485	0.36
*Actg1*	75/785	1.87	413/485	0.35
*Mat2a*	112/785	1.34	430/485	0.35
*Igfbp7*	365/785	0.7	485/485	0.34
*Rpl6*	660/785	1.82	238/485	0.33
*H2-D1*	157/785	1.35	245/485	0.33
*Cav2*	738/785	0.39	473/485	0.33
*Ctsd*	765/785	0.26	468/485	0.33
*Prkch*	168/785	1.22	354/485	0.31
*Rhob*	731/785	0.33	477/485	0.31
*Eef1g*	719/785	0.27	383/485	0.3
*Foxn3*	325/785	0.84	450/485	0.29
*Mmp15*	739/785	0.28	470/485	0.29
*Rpl10a*	117/785	1.4	398/485	0.28
*Eef2*	521/785	0.52	449/485	0.28
*Pecam1*	598/785	0.41	467/485	0.27
*Rps6*	48/785	2	396/485	0.26
*Oaz1*	592/785	0.65	432/485	0.26
*Gpi1*	750/785	0.36	407/485	0.26
*Ube2k*	751/785	0.25	389/485	0.26

DEGs from each comparison (neonatal healthy vs. neonatal post-MI and adult healthy vs. adult post-MI) are ranked according to the average log_2_FC and adjusted *P*-values and the common DEGs between the two comparisons were listed below, ordered based on the log_2_FC in adult mouse coronary ECs post-MI.

FC, fold change.

Notably, 752 (60.8%) genes were up-regulated in pro-regenerative (<P7) neonatal mouse coronary ECs but remained dormant in non-regenerative adult mouse coronary ECs post-MI (*Figure 3B*). We hypothesized that the reactivation of the expression of genes in this group may stimulate developmental regenerative pathways and, in turn, promote cardiac neovascularization in the post-ischaemic adult heart. *Vegfc* was selected to address this hypothesis, as it showed a high fold change in neonatal coronary EC after injury (0.47 log_2_FC), but minimal change in expression in adult EC post-MI (0.1 log_2_FC; *Figure 3C*). Further, the expression of VEGF-C receptors, *Flt4* and *Nrp2*, was up-regulated in both injured neonatal and adult ECs at various levels and timepoints (Supplementary material online, *Figure S2B*). We confirmed an increased expression of Flt4 and Nrp2 in the ischaemic vs. healthy heart using immunofluorescence staining (Nrp2^+^ vessel number *per* region = 43.0 ± 14.1 vs. 12.2 ± 4.1, *P* = 0.002, unpaired *t*-test; Flt4^+^ vessel number *per* region was 50.0 ± 30.8 in the infarct border region of the ischaemic heart, whereas no positively stained vessels or cells were observed in the healthy mouse heart; *Figure 3D* and *E*). The expression of both receptors was predominantly localized to the infarct border region, which is a known site of active neovascularization post-MI.^27^ To further investigate whether exogenous VEGF-C treatment would augment endogenous neovascularization in the adult mouse heart, *Pdgfb-iCreER^T2^-R26R-Brainbow2.1* multispectral lineage-tracing mice^27^ were administered tamoxifen to induce Cre-recombination and the stochastic expression of a yellow, red, green, or cyan fluorescent protein (YFP, RFP, GFP, or CFP) specifically in vascular ECs. Following a 14-day tamoxifen washout period, mice were then given recombinant human VEGF-C (rhVEGF-C) *via* intraperitoneal injection at Days 0, 2, 4, and 6 post-MI, which was induced by permanent ligation of the left anterior descending coronary artery^27^ (Supplementary material online, *Figure S2C*). At 7 days post-MI, the volume of fluorescent vascular clones derived following expansion of resident coronary ECs was quantified to reveal a significant increase in neovascularization in the infarct border in rhVEGF-C-treated hearts compared with the PBS-treated controls [clone volume (μm^3^) = 3072 ± 491.2 vs. 426 ± 105, *P* = 0.02; unpaired *t*-test]. A clone was defined as two or more adjacent ECs expressing the same *Brainbow2.1* fluorophore (*Figure 3F* and *G*). This demonstrates that exogenous VEGF-C treatment promotes vascular regeneration post-MI through direct activation of endogenous mechanisms of coronary EC clonal proliferation in the adult mouse heart.

### 3.4 Temporal analysis of the cardiac endothelial transcriptome in the adult mouse heart at early and late timepoints post-MI

**Figure 4:**
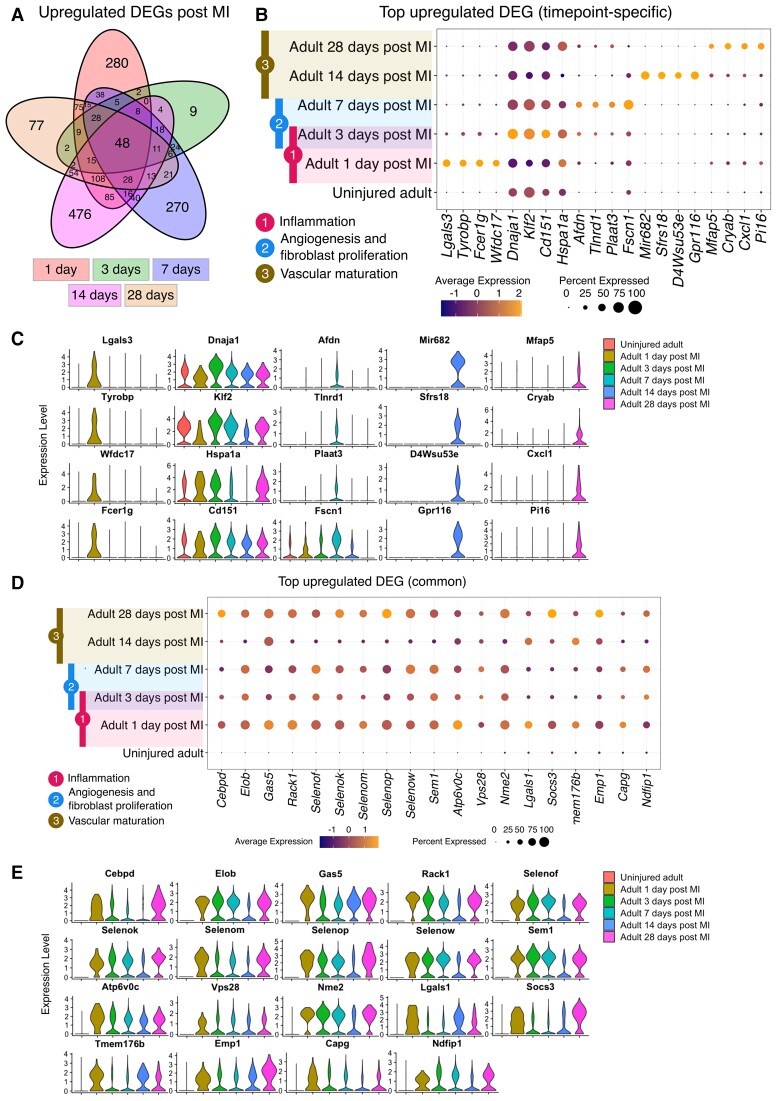
Temporal changes in cardiac endothelial transcriptome in the adult mouse heart after ischaemic injury. (*A*) Coronary EC scRNA-seq data from published studies of healthy and injured adult mouse hearts at Days 1, 3, 7, 14, and 28 post-MI were integrated and DEG analyses performed to identify transcriptomic changes specific to individual timepoints and common across all timepoints. (*B*) Top up-regulated DEGs at each timepoint post-MI with coloured bars indicating the three defined regenerative phases in mice (four DEGs were plotted for each stage with the ones mentioned in the main text indicated in boxes). (*C*) Violin plots showing top up-regulated DEGs at each timepoint post-MI. (*D*) Top up-regulated DEGs common across all timepoints post-MI compared with healthy cardiac ECs. (*E*) Violin plots showing top up-regulated DEGs common across all timepoints post-MI compared with healthy.

Coronary endothelial scRNA-seq data from independent published studies of healthy adult mouse hearts and at Days 1, 3, 7, 14, and 28 post-MI were integrated and analysed to identify the programmes that underpin timepoint-specific responses to ischaemic injury. Adult mouse coronary ECs from each timepoint were compared with uninjured ECs (*Figure 4A–C* and *Table 4*). This revealed DEGs that were enriched during defined regenerative and remodelling response phases after MI.^37^ For example, genes, *Lgals3* (1 day), *Tyrobp* (1 day), *Dnaja1* (3 days), and *Klf2* (3 days) showed increased expression in ECs during the inflammatory phase (3 h—3 days post-MI); *Dnaja1* (3 days), *Klf2* (3 days), *Afdn* (7 days), and *Tlnrd1* (7 days) were increased during the angiogenesis and fibroblast proliferation phase (2–7 days post-MI); and *Mir682* (14 days) and *Sfrs18* (14 days) were expressed during the vascular maturation phase (7–21 days).^37^ Moreover, 48 up-regulated genes were identified that were expressed by ECs at all injury timepoints and thus may also represent targets of potential interest for further experimental interrogation (*Figure 4D* and *E* and Supplementary material online, *Table S6*).

**Table 4:** Timepoint-specific DEGs up-regulated in adult mouse coronary endothelial cells post-MI

Days post-MI	Timepoint-specific up-regulated DEGs (average log_2_-fold change)
1	*Ptgs2* (1.72), *Ccl7* (1.93), *Lgals3* (3.07), *Wfdc17* (2.68), *Ctss* (2.77), *Tyrobp* (2.98), *Fcer1g* (2.79), *Il1b* (2.21), *Ccl9* (2.14), *Lilr4b* (1.77)
3	*Dnaja1* (1.02), *Klf2* (0.77), *Cd151* (0.60), *Psap* (0.53), *Hspa1a* (0.66), *Tuba1b* (0.46), *Ahsa1* (0.51), *Ptges3* (0.46), *Xbp1* (0.54)
7	*Afdn* (1.57), *Gm11361* (1.25), *Tlnrd1* (1.37), *Plaat3* (1.32), *Fscn1* (1.35), *Sox18* (1.18), *Inka1* (1.14), *Kctd17* (1.08), *Zfp979* (1.06), *Schip1* (0.98)
14	*Mir682* (3.45)*, Sfrs18* (2.42)*, D4Wsu53e* (1.83)*, Rab1* (1.36)*, Ppap2b* (1.77)*, Col1a1* (1.77)*, Orc5* (2.00)*, Cyb5* (1.10)*, Ccdc55* (1.19)*, Tenc1* (1.20)
28	*Fam71a* (1.40), *Cryab* (2.73), *Nbl1* (1.74), *Mfap5* (2.29), *Sdc1* (1.15), *Cxcl1* (3.12), *Sulf1* (1.02), *Rgs16* (1.56), *Pdlim2* (0.97), *Pi16* (2.60)

DEGs were ranked according to the average log_2_FC and adjusted *P*-values.

MI, myocardial infarction.

### 3.5 Analysis of foetal and adult human coronary vascular EC data sets reveals eight transcriptionally distinct cell clusters

**Figure 5:**
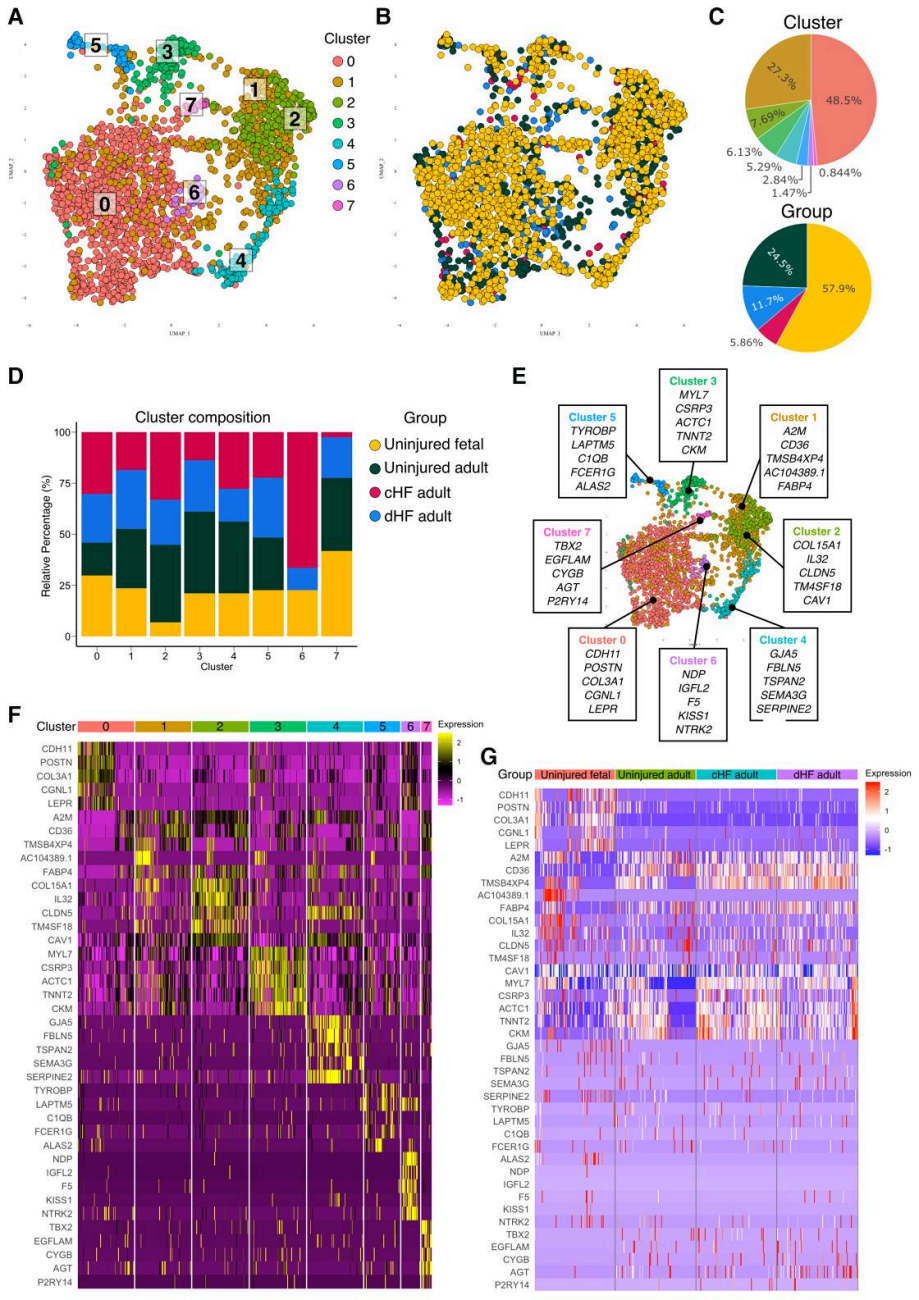
A single-cell transcriptomic map of human cardiac endothelial cells. (*A*) UMAP plot of 2251 human coronary ECs extracted from public transcriptomic data sets where each dot represents a single cell. Unsupervised clustering identified eight heterogenous EC clusters in the uninjured and injured foetal and adult human heart. Cells are colour coded for each associated EC state. (*B*) The cells are colour coded for the four different groups, including uninjured foetal, uninjured adult, cHF adult, and dHF adult. (*C*) Top, the percentage of ECs in each cluster; bottom, the percentage of ECs from each group. (*D*) Relative percentage of ECs from each cluster across all groups to resolve cluster composition. (*E*) Top expressed genes for each cluster identified *via* DEG analysis, comparing each cluster against the remaining ECs. The top five expressed genes for each cluster are provided. (*F*) Heatmap showing the expression of top cluster markers listed in *E* for each cluster. (*G*) Heatmap showing the expression of the top markers in each group. cHF, heart failure caused by ischaemic cardiomyopathy; dHF, heart failure caused by dilated cardiomyopathy.

scRNA-seq data sets generated from studies of primary human cardiac cells were curated from public repositories and pre-processed as described previously. ECs were extracted based on a panel of endothelial markers to form endothelial-specific data sets (Supplementary material online, *Figure S2D*). Unsupervised clustering was undertaken on 2251 human coronary vascular ECs (*Figure 5A*) extracted from five scRNA-seq data sets from four different groups, i.e. healthy foetal and adult human heart and from patients with heart failure caused by ICM (cHF) or dilated cardiomyopathy (dHF; Supplementary material online, *Table S1* and *Figure S1A*). Eight clusters (0–7) were identified containing ECs from each group distributed in varying proportions (*Figure 5A–D* and *Table 5*), thereby confirming successful integration of data from independent studies. GO-term analysis revealed the putative function of each cluster (Supplementary material online, *Table S7*), and DEG analysis was undertaken to reveal the top expressed genes by ECs in each cluster (*Figure 5E* and *F*). This showed, for example, that cells in Clusters 1 and 2 were associated with angiogenesis, and those in Clusters 5 and 6 were related to inflammatory responses. The expression of the top cluster markers was also examined in each group (*Figure 5G*).

**Table 5:** Human coronary endothelial cell cluster composition analysis

Cluster	Uninjured		Injured	
	Foetal human coronary ECs (%)	Adult human coronary ECs (%)	cHF adult coronary ECs (%)	dHF adult coronary ECs (%)
0	30.2	23.9	16.1	29.8
1	18.5	29.0	29.0	23.5
2	33.0	22.1	38.1	6.8
3	13.8	25.2	39.9	21.1
4	27.8	16.0	35.2	21.0
5	22.2	29.4	25.8	22.6
6	66.5	10.9	0	22.6
7	2.4	20.0	35.8	41.8

The percentage (%) of ECs in each cluster was calculated for each group and normalized across all groups.

ECs, endothelial cells; cHF, heart failure caused by ischaemic cardiomyopathy; dHF, heart failure caused by dilated cardiomyopathy.

### 3.6 Gene signatures expressed by coronary ECs in patients with heart failure with distinct aetiologies

**Figure 6:**
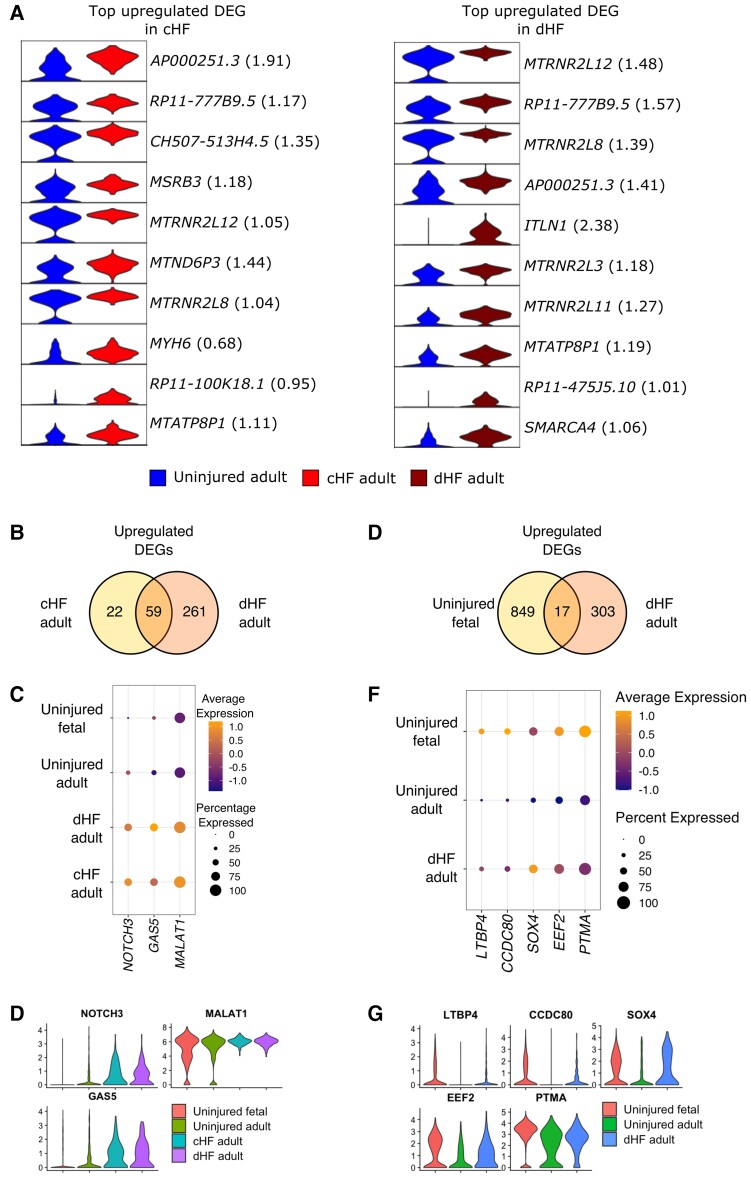
Comparative study of cardiac endothelial cell transcriptomes in patients with heart failure of different aetiologies. (*A*) Left, top up-regulated DEGs in coronary ECs in patients with cHF vs. the uninjured adult heart with log_2_FC details; right, top up-regulated DEGs in coronary ECs in dHF vs. the uninjured adult heart with log_2_FC details. (*B*) Up-regulated DEGs specific to each type of heart failure (cHF: 22; dHF: 261) and those common to both (59). (*C*) Top up-regulated DEGs common to both cHF and dHF coronary ECs compared with the uninjured heart. (*D*) Violin plots showing expression of *NOTCH3*, *GAS5*, and *MALAT1*. (*E*) Up-regulated DEGs specific to uninjured foetal and dHF coronary ECs, respectively (uninjured foetal: 849; dHF adult: 303), compared with healthy adult ECs, and ECs common to both (17). (*F*) Top up-regulated DEGs that are enriched in coronary ECs in the uninjured foetal heart, have low expression in the healthy adult heart, and are (re)up-regulated in the injured adult heart, specifically in patients with heart failure caused by dilated cardiomyopathy, indicating that developing mechanisms may be reactivated in the adult heart after injury. (*G*) Violin plots showing expression of *LTBP4*, *CCDC80*, *SOX4*, *EEF2*, and *PTMA*. cHF, heart failure caused by ischaemic cardiomyopathy; dHF, heart failure caused by dilated cardiomyopathy.

We identified the top up-regulated DEGs expressed by foetal vs. adult healthy human coronary ECs (Supplementary material online, *Table S8*), which likely play a role in human coronary vessel development and become quiescent in the adult heart. However, unlike our equivalent analyses in mice, a regenerative function of these genes cannot be inferred due to a lack of data from the injured foetal human heart. Gene signatures in ECs from patients with heart failure caused by ICM (cHF) or dilated cardiomyopathy (dHF) were then compared with those expressed by healthy adult human heart ECs to reveal similarities and differences in the endothelial transcriptome in patients with heart failure with differing aetiologies (*Figure 6A*). Interestingly, the long non-coding RNAs *AP000251.3* and *CH507-513H4.5* were among the top up-regulated endothelial markers in patients with cHF (1.91 and 1.35 log_2_-fold -change, respectively; *Figure 6A*). Intelectin-1/Omentin-1 (*ITLN1*) had the highest log_2_-fold change (2.38) among the top up-regulated genes in dHF compared with the healthy human coronary ECs, signifying a potential role in disease pathogenesis or vascular responses to injury. We identified 22 and 261 DEGs that were uniquely expressed by coronary ECs from either cHF or dHF patients, respectively, and 59 genes expressed by cells from both heart failure groups, compared with the healthy heart EC group (*Figure 6B*–*D* and Supplementary material online, *Table S9*). Among the commonly up-regulated genes were *NOTCH3* and the long non-coding RNAs, *GAS5* and *MALAT1*.

### 3.7 Reactivation of foetal gene expression in coronary ECs from patients with heart failure

To investigate whether foetal coronary developmental programmes were reactivated in the adult human heart endothelium in ischaemic disease, we compared DEGs in coronary ECs from the healthy foetal and adult human heart (Supplementary material online, *Table S8*) with those from patients with heart failure (Supplementary material online, *Table S9*). Seventeen genes were expressed at high levels in coronary ECs from the foetal heart and in patients with heart failure caused by dilated cardiomyopathy, but at low levels in ECs from the uninjured adult human heart (*Figure 6E*–*G* and Supplementary material online, *Table S10*) inferring reactivation of defined genes or gene signatures in disease. No genes were commonly enriched between the foetal heart EC group and patients with heart failure caused by ICM.

### 3.8 *Klf4*, *Egr1*, and *ZFP36* expression is induced during endogenous neovasculogenic responses in the mouse and human heart

**Figure 7:**
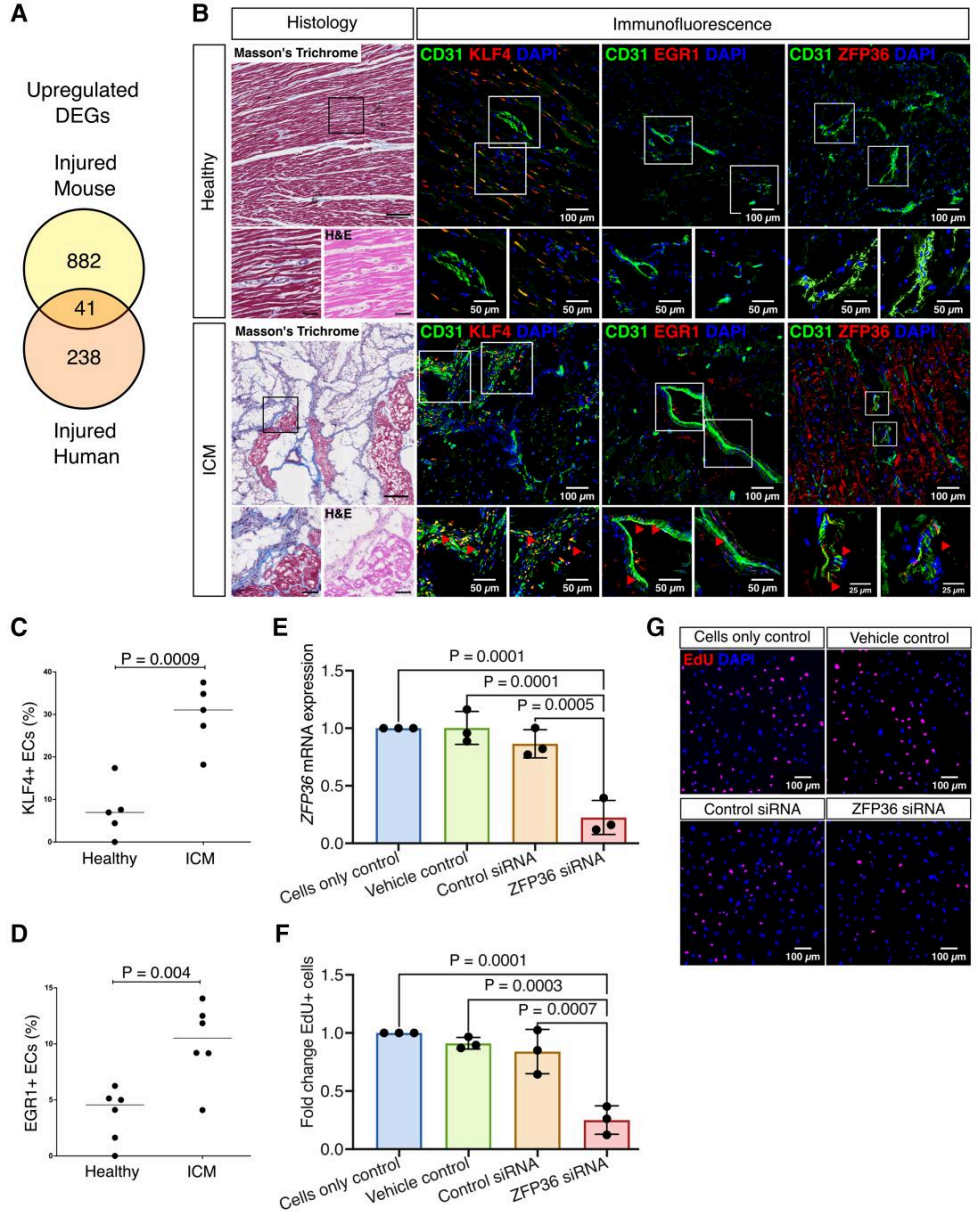
KLF4, EGR1, and ZFP36 play potential roles in endogenous neovasculogenic responses in the mouse and human heart after ischaemic injury. (*A*) All mouse and human coronary EC scRNA-seq data were integrated to identify conserved mechanisms underpinning vascular responses between the two species. Forty-one up-regulated DEGs were shared by coronary ECs in the injured mouse and human heart compared with the healthy hearts. However, many DEGs were specific to one species (882 and 238 genes, respectively). (*B*) Left, representative low-power images of serial sections from the healthy human heart and from patients with ischaemic cardiomyopathy stained using Masson’s Trichrome. The black boxes indicate the regions shown in the high-power images stained with both Masson’s Trichrome and H&E. Scale bar: 200 μm (low-power images), 50 μm (high-power images). Representative images of immunofluorescence for CD31 and KLF4(middle left)/EGR1(middle right)/ZFP36 (right), with DAPI for nuclei counterstain. The white boxes indicate the regions shown in the high-power images. ICM, ischaemic cardiomyopathy. Scale bar: 100 μm (low-power images), 50 and 25 μm (high-power images). (*C*) KLF4 expression was significantly increased in cardiac ECs in patients with ischaemic cardiomyopathy compared with the healthy human heart (% KLF4^+^ CD31^+^ EC = 29.7 ± 7.5% vs. 7.3 ± 6.4%, *P* = 0.0009; unpaired *t*-test). (*D*) EGR1 expression was significantly increased in cardiac ECs in patients with ICM compared with the healthy human heart (% EGR1^+^ CD31^+^ EC = vs. 10.1 ± 3.5% vs. 3.4 ± 2.5%, *P* = 0.004; unpaired *t*-test). (*E*) siRNA gene silencing of *ZFP36* in HCMECs gave a significant reduction at the mRNA level compared with the control siRNA (RQ = 0.86 ± 0.12 vs. 0.22 ± 0.15, *P* = 0.0005). (*F*) Proliferation assessed using an EdU incorporation assay was significantly inhibited following *ZFP36* gene silencing compared with control siRNA treatment (Fold change of %EdU^+^ human cardiac microvascular endothelial cells = 0.84 ± 0.19 vs. 0.25 ± 0.12, *P* = 0.0007). (*G*) Representative images of EdU stain on siRNA-treated HCMECs.

We next identified DEGs that were common to both the mouse and human coronary EC data sets after ischaemic injury. All mouse and human data sets were integrated, with only genes that satisfied one-to-one pairwise orthology included in the combined data set. Forty-one commonly up-regulated genes were identified in both human and mouse cardiac ECs after injury, irrespective of age, disease aetiology, or timepoint post-MI (*Figure 7A* and Supplementary material online, *Table S11*). *Klf4*, *Gas6*, and *Tsc22d3* showed consistent up-regulation at all timepoints studied post-MI in the adult mouse heart, and in both types of heart failure in human patients (Supplementary material online, *Figure S3A*). Other Klf family members were up-regulated in coronary ECs in the adult mouse and human heart following injury. As well as *Klf4*, *Klf6*, and *Klf9* showed an enriched expression in cells from patients with heart failure compared with the healthy human heart (Supplementary material online, *Figure S3B*). *Klf4* [Kruppel-like factor 4 (KLF)] was selected for validation due to its consistent increase across both species after injury (Supplementary material online, *Figure S3C*). We quantified KLF4 expression in the coronary endothelium in patients with ICM using immunofluorescence staining for KLF4 and CD31 (EC marker). This confirmed that KLF4 expression was significantly increased in the coronary vasculature of patients compared with healthy subjects, validating our gene expression data at the protein level, and highlighting a potential role of KLF4 in regulating endothelial activation post-MI (% KLF4^+^ CD31^+^ ECs = 29.7 ± 7.5% vs. 7.3 ± 6.4%, *P* = 0.0009; unpaired *t*-test; *Figure 7B* and *C*). Similar to *Klf4, Egr1* [early growth response 1 (EGR1)], and *Zfp36* (ZFP36 ring finger protein) also showed significant up-regulation in both mouse and human vascular ECs after ischaemic injury (Supplementary material online, *Figure S3C*). To validate these findings at the protein level, tissue sections from healthy human hearts and patients with ICM were stained using EGR1 and CD31 antibodies for quantitative analysis of coronary endothelial-specific EGR1 expression. EGR1 expression was significantly increased in coronary ECs in ICM patients (% EGR1^+^ CD31^+^ ECs = 10.1 ± 3.5% vs. 3.4 ± 2.5, *P* = 0.004; unpaired *t*-test; *Figure 7B* and *D*). Co-stain ZFP36 and CD31 showed high expression of ZFP36 protein in the ECs of the ICM tissue sections, especially in the intermediate filament, while minimal expression was found in the healthy controls (*Figure 7B*). We further carried out *in vitro* siRNA knockdown of *ZFP36* in HCMECs isolated from three individual human patients and assessed the impacts of *ZFP36* knockdown on EC proliferation using the EdU incorporation assay (*Figure 7E–G*). The *ZFP36* mRNA level was greatly reduced following the siRNA knockdown compared with control siRNA (RQ = 0.86 ± 0.12 vs. 0.22 ± 0.15, *P* = 0.0005), and cell proliferation was significantly inhibited following the *ZFP36* knockdown compared with the control siRNA treatment (fold change of %EdU^+^ HCMECs = 0.84 ± 0.19 vs. 0.25 ± 0.12, *P* = 0.0007).

### 3.9 CrescENDO, a Shiny app supporting exploration of EC gene expression data

We have developed a Shiny application ‘CrescENDO’ (www.crescendo.science) to support wider research communities to analyse gene expression data in coronary ECs in both mouse and human hearts at developmental and adult stages and spanning healthy and ischaemic conditions. CrescENDO contains the integrated data sets for mouse, human, and both combined, and allows researchers to select species and the specific data sets for exploration. It allows rapid analysis and visualization of the expression of multiple genes, indicating both the percentage of cells expressing the gene and the average expression level.

## 4. Discussion

We have undertaken high-dimensional characterization of mouse and human endothelial single-cell transcriptomic signatures, as they adapt throughout developmental and adult stages, and in response to ischaemic injury and disease. We have generated a framework to extract high-quality endothelial-specific data from studies of the whole cardiac cellulome, and for the integration of multiple scRNA-seq data sets generated in independent studies to allow broad-scope informatics meta-analyses. We present a publicly available comprehensive resource atlas, crescENDO, and envisage that this will foster future targeted studies of gene expression signatures in the coronary endothelium for a deeper understanding of the mechanisms associated with cardiovascular development and responses to ischaemic injury. We selected four targets, VEGF-C, Klf4, Egr1, and Zfp36, and validated their expression levels. Further, we experimentally demonstrate a function for ZFP36 in regulating coronary endothelial proliferation and show that VEGF-C administration enhances neovascularization *in vivo*.

This study aimed to tackle two critical issues currently faced in myocardial regenerative medicine. First, how can we improve translation of promising results observed in pre-clinical studies to the clinic? To address this, we undertook an unbiased analysis of data generated in independent studies in mice that have used an established MI model of coronary artery ligation^27,30–32^ and aligned this with single-cell data from patients with ischaemic cardiovascular disease.^36^ This identified targets conserved between species to thereby heighten translational significance and, importantly, confirmed the relevance and importance of mouse models to study human disease. We observed an increased expression of Klf4, a zinc finger transcription factor of the KLF family, in coronary vascular endothelium of both the injured mouse and human hearts compared with healthy heart endothelium. We further validated this finding at the protein level in cardiac tissues from patients with ICM compared with the healthy heart. A key regulatory role for KLF4 in vascular function has been shown *in vitro* and *in vivo*^38–41^ and Klf4 deficiency is associated with atherothrombosis,^42^ pulmonary arterial hypertension,^43^ and cerebral cavernous malformations^44^ in mice. Furthermore, inducible endothelial-specific *Klf4* knockout mice showed enhanced neointimal formation by Day 21 post-MI due to vascular smooth-muscle cell proliferation and inflammatory cell recruitment.^45^ Here, we report a further potential role for KLF4 in regulating endogenous neovasculogenic responses by ECs in the diseased adult human heart, lending credence to the conjecture that augmenting KLF4 may provide therapeutic benefit in patients with vascular disease.

Second, we aimed to identify pathways driving coronary vascular development that are redeployed in the injured adult heart and may therefore aid the advancement of regenerative therapies. We selected VEGF-C for further study due to its high expression in the developing mouse heart vasculature post-MI compared with the healthy heart. Specification and formation of the coronary vasculature and lymphatic system occur in synchrony during embryogenesis, principally mediated by VEGF-C signalling through VEGFR3.^46,47^ VEGF-C is often considered a selective growth factor for lymphatic EC in adult tissues, although multiple studies have reported VEGFR3 expression on adult vascular EC.^48–51^ Indeed, a potent angiogenic effect of VEGF-C in postnatal tissues was reported over 20 years ago^52^ and subsequent studies have shown regulation of both angiogenesis and lymphangiogenesis by VEGF-C in adult tissues.^50,53–57^ Here, we show that, in addition to a well-characterized role in modulation of lymphangiogenesis during myocardial regeneration and improved cardiac function,^58^ administration of exogenous VEGF-C can also significantly up-regulate neovascularization *via* endogenous coronary EC clonal proliferation in the infarct border region of the adult mouse heart post-MI. Future studies should investigate a potential dual regulatory role of VEGF-C in regulating both neovasculogenic and lymphangiogenic responses and associated downstream mechanisms, and how this can most effectively be translated to a clinical setting.

EGR1, a transcription factor involved in multiple cardiovascular diseases, vascular dysfunction, and inflammatory disorders,^59^ is enriched in the coronary ECs in both injured human and mouse hearts. Reducing EGR1 expression has shown to impair microvascular EC proliferation, migration, and microtubule network formation,^60^ indicating a potential role in promoting vascular regeneration post injuries. ZFP36 ring finger protein (ZFP36), or tristetraprolin, was also up-regulated in the coronary ECs in the injured mouse and human hearts. ZFP36 is an RNA-binding protein and an mRNA decay factor. A number of studies have demonstrated that ZFP36 modulates inflammatory activities,^61–64^ including a study in primary human aortic ECs showing that ZFP36 can reduce expression of inflammatory cytokines *via* inhibiting transcriptional activation and direct binding to destabilize target mRNAs.^64^ ZFP36 also regulates cell cycle and VEGF production in keratinocytes,^65^ suggesting a potential role in angiogenesis. However, little is known about whether ZFP36 regulates vascular responses after ischaemic injuries, especially regenerative response. In this study, we observed prominent activation of *ZFP36* at protein level in the ischaemic human heart tissue compared with the other two targets *KLF4* and *EGR1*, which prompted us to pursue an *in vitro* siRNA silencing study of *ZFP36* in primary HCMEC lines to assess its impacts on EC proliferation. *ZFP36* siRNA silencing showed significant inhibition on cell proliferation, providing solid evidence that *ZFP36* regulates endothelial proliferation, making it a promising novel target for cardiovascular regeneration. Considering its essential role in inflammatory activity regulation, *ZFP36* may elicit protective effects in the heart through a multi-faceted manner after ischaemic injuries.

We have incorporated high-quality single-cell RNA-seq data from selected studies of the mouse and human heart published between 2018 and 2021. This has identified unique gene expression profiles in the vasculature of patients with heart failure caused by either dilated cardiomyopathy or by ICM. This insight into key differences in the mechanisms underlying cell responses in different types of heart failure supports the conjecture that disease-specific treatment may be warranted.^36^ We further identified DEGs common to the endothelium in foetal human heart and patients with heart failure due to dilated cardiomyopathy. These may represent relevant targets for future strategies to enhance myocardial angiogenesis, supported by recent data from other groups. For example, PTMA was shown to drive angiogenesis and improve cardiac function after ischaemic injury in the adult mouse heart and PABPC1 has been implicated in both vascular development and as potentially advantageous in the failing or dilated myocardium.^66^ Therefore, future interrogation of the effects on endogenous angiogenesis may reveal the relevance of these findings for the treatment of patients with heart failure.

A potential limitation of our approach is a lack of data from patients with acute MI. Therefore, we undertook protein level target validation on cardiac tissues from patients with acute ischaemic disease to ensure relevance during early stages of disease pathogenesis, since prompt intervention is critical to promote vascular perfusion and salvage injured ischaemic myocardium. Moreover, as single-cell RNA-sequencing technology continues to evolve at an unprecedented rate, we have generated a crescENDO app to permit addition of new published data sets and ensure that ongoing and future analyses are of the most relevant and current data.

## 5. Conclusion

We present a comprehensive high-resolution meta-analysis of a wealth of independent single-cell RNA-sequencing data that has emerged over recent years from studies of the mouse and human heart. This has characterized endothelial heterogeneity during coronary development and temporal regenerative responses to injury. We have identified numerous specific novel targets with a potential role in mediating neovascularization and cardiac regeneration. Finally, we provide compelling new evidence that Klf4, VEGF-C, Egr1, and Zfp36 are critical regulators of endothelial responses in the diseased mouse and human adult heart. This study provides a deeper understanding of the molecular mechanisms that underpin cardiac regeneration and may inform future cardiac regenerative strategies for patients with heart disease.

## Supplementary material

Supplementary material is available at *Cardiovascular Research* online.

## Supplementary Material

cvac151_Supplementary_Data

## Data Availability

The data underlying this article are available in an interactive Shiny web application at http://www.crescendo.science, and the data sets used in the study are in the GEO, ArrayExpress, and SRA (Supplementary material online, *Table S1*).

## Notes

Translational perspective Myocardial infarction is the leading cause of heart failure, a condition with high morbidity and mortality. Vascular regeneration is vital to tissue survival and functional restoration while a comprehensive understanding of the underpinning mechanisms is lacking. We show that the meta-analysis of single-cell RNA-sequencing data excels at deciphering multi-faceted vascular responses conserved between mouse and human following ischaemic injury. We envision that these data will inform future strategies and accelerate therapeutic development for promoting vascular regeneration in the ischaemic heart.
