# Supplementary material for: Multi-species meta-analysis identifies transcriptional signatures associated with cardiac endothelial responses in the ischaemic heart
Source: Cardiovasc Res. 2022 Sep 9;119(1):136–54. doi: 10.1093/cvr/cvac151 (PMC10022865; doi:10.1093/cvr/cvac151)
Supplement: cvac151_Supplementary_Data [file cvac151_supplementary_data.docx]

**Supplementary Material**

**Multi-species meta-analysis identifies transcriptional signatures associated with cardiac endothelial responses in the ischaemic heart**

Ziwen Li^1^, Emmanouil G Solomonidis^1^, Bronwyn Berkeley^1^, Michelle Nga Huen Tang^1^, Katherine Ross Stewart^1^, Daniel Perez-Vicencio^1^, Ian R McCracken^1^, Ana-Mishel Spiroski^1^, Gillian A Gray^1^, Anna K Barton^1^, Stephanie L Sellers^2^, Paul R Riley^3^, Andrew H Baker^1^, Mairi Brittan^1^*

^1^Centre for Cardiovascular Science, The Queen’s Medical Research Institute, University of Edinburgh, Edinburgh EH16 4TJ, UK.

^2^Division of Cardiology, Cardiovascular Translational Lab, and Centre for Heart Lung Innovation, St Paul's Hospital and University of British Columbia, Vancouver, Canada.

^3^University of Oxford, Department of Physiology, Anatomy and Genetics, South Parks Road, Oxford OX1 3PT, UK.

*Corresponding author: Mairi Brittan ([mbrittan@ed.ac.uk](mailto:mbrittan@ed.ac.uk))

**Supplementary Tables**

**Supplementary Table 1. Datasets included in the study.** P, postnatal day; MI, myocardial infarction; cHF, heart failure caused by ischaemic cardiomyopathy; dHF, heart failure caused by dilated cardiomyopathy; ECs, endothelial cells; LV, left ventricle; LA, left atrium.

| Species | Repository | Repository accession number | Injury/Disease status | Age | Number of ECs post-filtering | References |
| --- | --- | --- | --- | --- | --- | --- |
| Mouse | GEO | GSE117893 | 21 days post-MI | Neonatal (P2) | 270 | Li et al., Development 2018^28^ |
|  | GEO | GSE118545 | Healthy | Neonatal (P6) | 1510 | Hu et al., Genes & Development 2018^29^ |
|  |  |  |  | Neonatal (P10) | 1211 |  |
|  | ArrayExpress | E-MTAB-7376 | 3 days post-MI | Adult | 519 | Farbehi et al., eLife 2019^30^ |
|  | GEO | GSE132880 | Healthy | Adult | 2747 | Li et al., EHJ 2019^27^ |
|  |  |  | 7 days post-MI |  | 5168 |  |
|  | GEO | GSE136088 | 14 days post-MI | Adult | 285 | DePasquale et al., Nuclei acids research 2019^31^ |
|  | ArrayExpress | E-MTAB-9816 | 1 day post-MI | Adult | 59 | Tombor et al., Nature Communications 2021^32^ |
|  |  |  | 3 days post-MI |  | 116 |  |
|  |  |  | 14 days post-MI |  | 169 |  |
|  |  |  | 28 days post-MI |  | 177 |  |
| Human | GEO | GSE106118 | Healthy | Fetal  5 - 25 weeks | 240 | Cui et al., Cell reports 2019^33^ |
|  | GEO | GSE134355 | Healthy | Fetal 11 - 12 weeks | 532 | Han et al., Nature 2020^34^ |
|  |  |  |  | Adult | 165 |  |
|  | SRA | SRP234812 | Healthy | Fetal  19 - 22 weeks | 532 | Suryawanshi et al., Cardiovascular research 2020^35^ |
|  | GEO | GSE109816 | Healthy | Adult | 386 | Wang et al., Nature cell biology 2020^38^ |
|  |  | GSE121893 | cHF | Adult | 132 |  |
|  |  |  | dHF |  | 264 |  |

**Supplementary Table 2. Technical metrics for the datasets included in the study.** P, postnatal day; MI, myocardial infarction; cHF, heart failure caused by ischaemic cardiomyopathy; dHF, heart failure caused by dilated cardiomyopathy.

| Repository accession number | Injury/Disease status | Platform | Type | Sequencing depth |
| --- | --- | --- | --- | --- |
| GSE117893 | 21 days post-MI | 10X Chromium v2 | Single nuclei | **Rep1**: 3,505 cells; 36,573 reads per cell; 548 genes per cell; 72.4% saturation  **Rep2**: 3,478 cells; 35,908 reads per cell; 546 genes per cell; 72.0% saturation  **Rep3**: 3,514 cells; 36,793 reads per cell; 549 genes per cell; 72.6% saturation  **Rep4**: 3,503 cells; 36,634 reads per cell; 549 genes per cell, 72.5% saturation |
| GSE118545 | Healthy P6/P10 | Drop-Seq | Single nuclei | Sequencing depth is not reported. Post EC extraction: 702 median genes per cell for P6 and 881 median genes per cell for P10. |
| E-MTAB-7376 | 3 days post-MI | 10X Chromium v2 | Single cells | **Rep1**: 7,515 cells; 30,057 reads per cell; 978 genes per cell; 80.3% saturation |
| GSE132880 | Healthy | 10X Chromium v2 | Single cells | **Rep1**: 2,962 cells; 98,063 reads per cell; 1154 genes per cell; 94.1% saturation |
|  | 7days post-MI | 10X Chromium v2 | Single cells | **Rep1**: 6,298 cells; 48,079 reads per cell; 1713 genes per cell; 83.1% saturation |
| E-MTAB-9816 | 1 day post-MI | 10X Chromium v3 | Single cells | **Rep1**: 1,176 cells; 159,511 reads per cell; 1,467 genes per cell; 92.7% saturation |
|  | 3 days post-MI | 10X Chromium v3 | Single cells | **Rep1**: 1,405 cells; 142,018 reads per cell; 1,785 genes per cell; 89.2% saturation |
|  | 14 days post-MI | 10X Chromium v3 | Single cells | **Rep1**: 2,446 cells; 203,484 reads per cell; 1,266 genes per cell; 96.3% saturation |
|  | 28 days post-MI | 10X Chromium v3 | Single cells | **Rep1**: 3,213 cells; 132,482 reads per cell; 1,248 genes per cell; 95% saturation |
| GSE106118 | Healthy | SMART-seq2 | Single cells | ~ 3,700 genes/cell, ~ 100,000 reads/cell |
| GSE134355 | Healthy fetal | Microwell-seq | Single cells | **Rep1**: 5319 cells; 717 genes/cell  **Rep2**: 2678 cells; 942 genes/cell |
| GSE134355 | Healthy adult | Microwell-seq | Single cells | **Rep1**: 1,308 cells; 701 genes/cell  **Rep2**: 1478 cells; 808 genes/cell |
| SRP234812 | Healthy fetal | 10X Chromium v2 | Single cells | **Rep1**: 4,120 cells; 49,515 reads per cell; 1231 genes per cell; 80.3% saturation  **Rep2**: 4,739 cells; 34,093 reads per cell; 1,174 genes per cell; 71.8% saturation  **Rep3**: 5,381 cells; 28,856 reads per cell; 883 genes per cell; 56.9% saturation |
| GSE109816 | Healthy adult | SMART-seq | Single cells | 294,652 reads per cell, 2,125 genes per cell |
| GSE121893 | HF adult (including cHF and dHF) | SMART-seq | Single cells | 378,969 reads per cell, 1,983 genes per cell |

**Supplementary Table 3. Details of patients included in the KLF4 validation study**.

|  | **Age** | **Sex** | **Cause of death** |
| --- | --- | --- | --- |
| **Healthy subjects**  **(n = 5)** | 25 | Male | 1a - Suspension by ligature |
|  | 52 | Male | 1a Chest injuries, 1b Road traffic collision (cyclist) |
|  | 29 | Male | 1a - Suspension by ligature |
|  | 53 | Male | 1a - Suspension by ligature |
|  | 63 | Male | 1a - Ruptured atherosclerotic abdominal aortic aneurysm |
|  |  |  | **Diagnosis** |
| **Patients**  **(n = 5)** | 45 | Female | Ischaemic cardiomyopathy |
|  | 70 | Male | Ischaemic cardiomyopathy |
|  | 52 | Male | Ischaemic cardiomyopathy |
|  | 55 | Male | Ischaemic cardiomyopathy |
|  | 57 | Female | Ischaemic cardiomyopathy |

**Supplementary Table 4. Details of *ZFP36* siRNA oligonucleotides**.

| siRNAs | Sequence (5’ -> 3’) | |
| --- | --- | --- |
|  | Sense | Antisense |
| s14977 | UCUGUCUCCUAGAAUCUUAtt | UAAGAUUCUAGGAGACAGAtt |
| s14978 | AGACGGAACUCUGUCACAAtt | UUGUGACAGAGUUCCGUCUtg |
| s14979 | CUUUAUUUAUGACGACUUUtt | AAAGUCGUCAUAAAUAAAGgg |

**Supplementary Table 5.** **Top marker and GO term analyses of mouse coronary endothelial cell clusters.** Genes enriched in individual clusters were identified as the cluster markers. The markers were ranked by the adjusted *P* values and average log2FC and the top 20 markers that satisfied adjusted *P* < 0.05 were listed below.

| Cluster | Top markers | Top GO terms |
| --- | --- | --- |
| 0 | *Rps6, Rpl18a, Rps28, Rpl13a, Rps7, Rps8, Rps23, Rpl34, Rpl37, Rps27, Rps4x, Rpl26, Rps12, Ttn, Id3, Hsp90aa1, Rps21, Tm4sf1, Cyyr1* | Positive regulation of inflammatory response, regulation of transcription involved in cell fate commitment, protein transport from ciliary membrane to plasma membrane |
| 1 | *Actb, Trp53i11, Mest, Tmsb10, Prnd, Sparcl1, Tmsb4x, Ptp4a3, Pfn1, Tpm4, Myl6, Actg1, Csrp1, Ppp1r2, Msn, Bst2, Ubb, Mall, Ly6a, Hspa1a* | Cytoplasmic translation, ribosomal small subunit assembly, sequestering of actin monomers |
| 2 | *Cxcl12, Aqp7, Cd300lg, Btnl9, Cd36, Sept4, Slc6a6, Cdkn1c, Oaz2, Glul, Car4, Clec14a, Flt1, Tubb2a, Gas6, App, Morf4l1, Eva1b, Dazap2* | Cell adhesion, positive regulation of angiogenesis, regulation of endothelial cell proliferation |
| 3 | *Aqp1, Igfbp5, Cd9, Rassf9, Rhoj, Fabp4, Slc26a10, Anxa2, Adgrf5, Ptrf, Dstn, Crip2, Rras, Lmo2, Prr13, Aplp2* | Positive regulation of fibroblast proliferation, negative regulation of epithelial cell apoptotic process, negative regulation of developmental process |
| 4 | *Jun, Btg2, Klf4, Klf2, Dusp1, Jund, Cdkn1a, Ubc, Apold1, Sgk1, Irf1, Hspb1, Plk2, Pnrc1, Atp2a2, Wsb1, Dusp3, Plat* | Negative regulation of apoptotic process, regulation of cell cycle, positive regulation of nitric oxide biosynthetic process |
| 5 | *Fbln5, Gja5, Hey1, Sox17, Ltbp4, Efnb2, Rbp7, Ly6c1, Depp1, Cyb5r3, Nudt4, Cd81, Emp2, Serinc3, Id2, Eif1, My12a* | Angiogenesis, positive regulation of epithelial cell proliferation, cell adhesion |
| 6 | *Stmn1, Hmgb2, Cdc20, Cenpf, Top2a, Ube2c, Hist1h2ae, Cenpa, Selenoh, Dek, Ube2s, Tuba1b, Tubb5, Calm2, Arl6ip1, Tubb4b, Ptma, H10, Ctla2a, Gm42418* | Regulation of alternative mRNA splicing, via spliceosome, mitotic sister chromatid segregation, mismatch repair |
| 7 | *Tmem59, Hspg2, Rabac1, Pdgfb, Scp2, Dusp3, Notch4, Itpkb* | Branching involved in blood vessel morphogenesis, angiogenesis, positive regulation of angiogenesis |
| 8 | *Cox6a2, Ankrd1, Tnni3, Pln, Actc1, Myl2, Tnnt2, Slc25a4, Tpm1, Timp3, Gm10250, Mdh1, Atp5g1, Heg1, Rps26, Romo1* | Cytoplasmic translation, ribosomal small subunit assembly, sequestering of actin monomers |
| 9 | *Plvap, Lrg1, Tmem176a, Tmem176b, Cpe, Cfh, Socs3, Selenop, Rplp0, Rps2, Rpl15, Ifitm2, Scarb1, Itm2b, Slc25a5, Ebf1, Rgcc, Igfbp7, Fos* | Cytoplasmic translation, ribosomal small subunit assembly, positive regulation of angiogenesis |
| 10 | *Rbm20, Slc8a1, Ctnna3, Myocd, Gm26561, RP24-559D16.1, Ryr2, Palld, Fhl2, Ccdc141, Chrm2, Dmd, Kcnd2, Pde4d, Pip5k1b, Sorbs2, Tbc1d4, Lmo7, Sorbs1, Klf6* | Regulation of inflammatory response, sequestering of metal ion, negative regulation of platelet-derived growth factor receptor-beta signalling pathway |
| 11 | *Col3a1, Col1a1, Dcn, Col1a2, Postn, Lum, Mfap5, Htra3, Gsn, Gpx3, Rarres2, Serping1, Rcn3, Pi16, Aebp1, Eef1a1, Mgp, Ncl, Crip1* | Cellular modified amino acid metabolic process, intrinsic apoptotic signalling pathway in response to DNA damage, small molecule biosynthetic process |
| 12 | *Ifit2, Cxcl10, Isg15, Gbp2, Gbp5, Gbp3, Rsad2, Oasl2, Iigp1, Isg20, Samhd1, Ifitm3, AW112010, Cd47, Plscr2, H2-D1, Chmp4b, Upp1, Clic4, Timp4* | Defence response to virus, cellular response to interferon-beta, positive regulation of T cell mediated cytotoxicity |
| 13 | *Ctss, Wfdc17, Cd14, C1qc, C1qa, Ccl4, C1qb, Lyz2, Cxcl2, Spp1, Pf4, Apoe, Cd74, H2-Aa, Ctsb, Fth1, H2-Ab1, Cst3, Ftl1, Srgn* | Cytoplasmic translation, positive regulation of signal transduction by p53 class mediator, immune system process |
| 14 | *Rgs4, Myh11, Higd1b, Gucy1a3, Vtn, Steap4, Ndufa4l2, Kcnj8, Gm13889, Rasl11a, Tpm2, Rgs5, Abcc9, Cox4i2, Myl9, Cox4i2, Myl9, Colec11, Axl, Lbh* | Transmembrane receptor protein serine/threonine kinase signalling pathway, hepatocyte proliferation, circadian behaviour |

**Supplementary Table 6. Common upregulated DEGs across all timepoints post-MI in adult mouse cardiac ECs compared to uninjured adult mouse cardiac ECs.** Differentially expressed genes (DEGs) were ranked according to the adjusted P values and the average log2 fold change.

| DEGs | Average log2 fold change | | | | |
| --- | --- | --- | --- | --- | --- |
|  | 1 day | 3 days | 7 days | 14 days | 28 days |
| *Cebpd* | 2.69 | 2.65 | 1.63 | 2.34 | 3.58 |
| *Elob* | 2.66 | 2.76 | 2.77 | 1.49 | 2.76 |
| *Gas5* | 3.77 | 2.74 | 2.50 | 3.22 | 3.50 |
| *Rack1* | 3.22 | 2.75 | 2.49 | 1.95 | 2.99 |
| *Selenof* | 2.70 | 2.79 | 3.03 | 1.64 | 2.62 |
| *Selenok* | 2.38 | 2.43 | 2.37 | 1.63 | 2.89 |
| *Selenom* | 2.39 | 1.93 | 2.02 | 1.40 | 2.35 |
| *Selenop* | 3.65 | 3.16 | 2.99 | 2.73 | 4.47 |
| *Selenow* | 3.04 | 2.92 | 3.36 | 2.20 | 3.28 |
| *Sem1* | 2.93 | 3.02 | 3.05 | 1.63 | 2.72 |
| *Atp6v0c* | 3.02 | 2.40 | 2.14 | 1.84 | 2.11 |
| *Vps28* | 1.15 | 1.51 | 1.57 | 1.19 | 1.30 |
| *Nme2* | 2.81 | 2.62 | 2.51 | 1.79 | 2.90 |
| *Lgals1* | 2.65 | 0.95 | 0.87 | 2.26 | 1.73 |
| *Socs3* | 1.99 | 1.17 | 0.76 | 1.87 | 3.16 |
| *Tmem176b* | 1.42 | 0.70 | 0.75 | 1.45 | 0.99 |
| *Emp1* | 1.22 | 1.19 | 1.18 | 1.44 | 2.43 |
| *Capg* | 1.40 | 1.16 | 1.15 | 0.46 | 0.87 |
| *Ndfip1* | 0.76 | 1.51 | 1.41 | 0.55 | 1.30 |

**Supplementary Table 7.** **Top marker and GO term analyses of human coronary endothelial cell clusters.** Genes enriched in individual clusters were identified as the cluster markers. The markers were ranked by the adjusted *P* values and average log2FC and the top 20 markers that satisfied adjusted *P* < 0.05 were listed below.

| Cluster | Top markers | Top GO terms |
| --- | --- | --- |
| 0 | *CDH11, POSTN, COL3A1, CGNL1, LEPR, SAT1, CLU, PROCR, FBLN2, CCDC80, NPR3, TIMP1, COLEC11, LTC4S, SLC6A4, FN1, APCDD1, CTHRC1, INHBA, NPPC* | Translational initiation, viral transcription, nuclear-transcribed mRNA catabolic process, nonsense-mediated decay, SRP-dependent cotranslational protein targeting to membrane, cytoplasm translation, ribosomal large subunit assembly, maturation of SSU-rRNA from tricistronic rRNA transcript, ribosomal small subunit assembly, heterophilic cell-cell adhesion via plasma membrane cell adhesion, calcium-dependent cell-cell adhesion via plasma membrane cell adhesion molecule |
| 1 | *A2M, CD36, TMSB4XP4, AC104389.1, FABP4, MTATP6P1, H19, FABP5P7, IGF2, RGCC, COL4A1, COL15A1, RP5-857K21.11, FMO2, KDR, MGLL, COL4A2, ITGA6* | Angiogenesis, maintenance of blood-brain barrier, vesicle transport along actin filament, negative regulation of dendrite development, negative regulation of endothelial cell proliferation, platelet aggregation, protein localisation to adherens junction, antigen processing and presentation of exogenous peptide antigen via MHC class I, TAP-independent, positive regulation of endothelial cell proliferation, positive regulation of GTPase activity |
| 2 | *COL15A1, IL32, CLDN5, TM4SF18, CAV1, COL4A1, GNG11, CA4, FABP5, LGALS1, IGFBP7, COL4A2, TNNT3, C1orf54, S100A16, GYPC, IGFBP2, IGFBP3, FABP4, CCND1* | Positive regulation of cell migration, angiogenesis, Fc-gamma receptor signalling pathway involved in phagocytosis, negative regulation of endothelial cell proliferation, vascular endothelial growth factor receptor signalling pathway, interleukin-12-mediated signalling pathway, basement membrane organisation, regulation of cell shape, postsynaptic actin cytoskeleton organisation, negative regulation of blood vessel endothelial cell migration |
| 3 | *MYL7, CSRP3, AcTC1, TNNT2, CKM, TPM1, MYL2, MYH7, CRYAB, TNNI3, NPPA, TNNC1, HSPB7, TTN, MB, COX6A2, ANKRD1, COX7A1, MYH6, AP000251.3* | Muscle filament sliding, cardiac myofibril assembly, sarcomere organisation, cardiac muscle contraction, mitochondrial ATP synthesis coupled electron transport, regulation of cardiac muscle contraction by regulation of the release of sequestered calcium ion, skeletal muscle thin filament assembly, cellular response to caffeine, atrial cardiac muscle cell action potential, membrane depolarisation during cardiac muscle cell action potential |
| 4 | *GJA5, FBLN5, TSPAN2, SEMA3G, SERPINE2, PCSKS, CLDN5, SRGN, JAG1, HEY1, CXCL12, MECOM, JAM2, CLEC14A, PPA1, IGFBP3, DKK2, KCTD12, ELN, SULF1* | Negative regulation of protein binding, platelet degranulation, extracellular matrix organisation, positive regulation of cell differentiation, heart trabecula formation, negative regulation of cell migration, protein localisation to bicellular tight junction, response to interferon-alpha, negative regulation of phosphoprotein phosphatase activity, blood vessel remodelling |
| 5 | *TYROBP, LAPTM5, C1QB, FCER1G, ALAS2, S100A9, LYZ, S100A8, PLAUR, CXCL8, DUSP2, HBM* | Neutrophil degranulation, respiratory burst, positive regulation of tumour necrosis factor production, innate immune response, positive regulation of interleukin-4 production, positive regulation of interleukin-6 production, microglial cell activation, positive regulation of interferon-gamma production, cytokine-mediated signalling pathway, neutrophil chemotaxis |
| 6 | *NDP, IGFL2, F5, KISS1, NTRK2, SELL, TFPI2, HAPLN1, PTHLH, PLAT, CD55, SERPINB1, AKR1B1, DKK2, CD9, S100A6, CRIP1, DKK3, IGFBP5* | Nuclear-transcribed mRNA catabolic process, nonsense-mediated decay, translational initiation, SRP-dependent cotranslational protein targeting to membrane, viral transcription, neutrophil degranulation, cytoplasmic translation, antigen processing and presentation of exogenous peptide antigen via MHC class I, TAP-dependent, antigen processing and presentation of exogenous peptide antigen via MHC class I, TAP-independent, positive regulation of translation, antigen processing and presentation of endogenous peptide antigen via MHC class I via ER pathway, TAP-independent |
| 7 | *TBX2, EGFLAM, CYGB, AGT, P2RY14, ITGB1P1, MYLK, PDGFRB, STEAP4, RGSS, SCN4B, NOTCH3, THBS4, FAT1, KLHL23, ABCC9* | Brain development, negative regulation of blood vessel endothelial cell migration, positive regulation of signal transduction by p53 class mediator, endothelial cell differentiation, peptide hormone processing, muscle contraction, branching morphogenesis of an epithelial tube, epithelial cell development, negative regulation of cellular process, negative regulation of cell-substrate adhesion |

**Supplementary Table 8. Upregulated DEGs in human fetal coronary ECs compared to adult ECs.** DEGs were ranked according to both the adjusted P values and the average log2 fold change.

| Comparison | Upregulated (log2 fold change) |
| --- | --- |
| Uninjured fetal vs uninjured adult | *HBG2* (6.24), *EEF1A1* (2.89), *RPL10* (2.77), *HBA1* (5.14), *HBA2* (5.25), *MDK* (3.47), *RPL15* (2.13), *RPS23* (2.31), *RPS26* (2.44), *GAPDH* (2.38), *RPS15A* (2.85), *RAMP2* (2.72), *RPL13*(2.34), *COL3A1* (3.88), *RPS7* (2.37), *RPS3A* (2.30), *RPL26* (2.55), *RPL39* (2.61), *RPLP0* (2.22), *RPL28* (2.67) |

**Supplementary Table 9. Gene expression in cardiac ECs from patients with heart failure, compared to those from the healthy adult human heart. Differentially expressed genes** (DEGs) were ranked according to the adjusted P values and the average log2 fold change. cHF, heart failure caused by ischaemic cardiomyopathy; dHF, heart failure caused by dilated cardiomyopathy.

| Groups | Upregulated (log2 fold change) | |
| --- | --- | --- |
|  | Specific | Common (cHF/dHF) |
| cHF | *TRMT61A* (0.38), *RNY4* (1.12), *TTN* (0.63), *ANKRD1* (0.63), *PDK4* (1.00), *DNAJC6* (0.67), *PCNXL3* (0.40), *TCEB2P2* (0.60), *ANKRD36C* (1.34), *RN7SKP71* (1.16) | *MSRB3* (1.18/1.04), *MYH6* (0.68/0.26), *MTATP8P1* (1.11/1.19), *SMARCA4* (0.83/1.06), *KANK2* (0.89/0.61), *MYL7*(0.52/0.39), *DOCK6* (0.74/0.85), *NOTCH3* (0.39/0.28), *SYNE3* (0.37/0.48), *CANX* (0.45/0.38), *GAS5* (0.29/0.43), *ONECUT3* (0.58/0.35), *PTMAP5* (0.75/0.89), *MALAT1* (0.41/0.38), *UACA* (0.80/0.32), *SCN5A* (0.44/0.35), *NBL1* (0.92/0.84), *WDR31* (0.49/0.66) |
| dHF | *ITLN1* (2.38), *RP11-475J5.10* (1.01), *HP* (1.20), *FOSB* (1.09), *MTND4P12* (0.90), *RP11-475J5.6* (0.84), *MTRNR2L7*, *RP5-857K21.6*, *MTRNR2L13*, *JUN* |  |

**Supplementary Table 10. Common upregulated DEGs in fetal and dHF adult heart ECs compared to the uninjured adult heart.** DEGs were ranked according to both the adjusted P values and the average log2 fold change.

| Comparison | Upregulated (log2 fold change, dHF/fetal) |
| --- | --- |
| Common DEGs between healthy fetal and dHF adult (vs uninjured adult) | *RPL39* (0.35/2.61), *RPL11* (0.36/1.91), *PTMA* (0.40/1.16), *RPS11* (0.37/1.19), *FN1* (0.25/2.23), *NAP1L1* (0.37/0.93), *EFNB2* (0.47/0.63), *RPL10AP6* (0.51/0.25), *LTBP4* (0.55/1.02), *CCDC80* (0.33/1.03), *SOX4* (1.08/0.63), *EEF2* (0.61/1.01), *HSPA5* (0.40/0.86), *FUS* (0.76/0.77), *PABPC1* (0.75/0.58), *RTF1* (0.27/0.43), *TIMP2* (0.39/0.29) |

**Supplementary Table 11. Common and species-specific upregulated genes in injured mouse and human cardiac ECs.** DEGs were ranked according to the adjusted P values and the average log2 fold change.

|  | Upregulated DEGs (log2 fold change, mouse/human) | |
| --- | --- | --- |
|  | Common | Species-specific |
| Mouse injured vs uninjured | *Cebpd*(1.75) /*CEBPD* (0.90), *Fos* (1.54)/*FOS* (0.52), *Jund* (0.82)/*JUND* (0.56), *Jun* (1.09)/*JUN* (1.62), *Rps4x* (0.69)/*RPS4Y1* (0.57), *Lgals3bp* (0.90)/*LGALS3BP* (0.35), *Cd74* (0.92)/*CD74* (0.66), *B2m* (0.67)/*B2M* (0.54), *Dnaja1* (0.75)/*DNAJB1* (1.15), *Zfp36* (1.20)/*ZFP36* (0.77), *Zfp36l2* (0.80)/*ZFP36L2* (0.79), *Adamts1* (1.05)/*ADAMTS1* (0.69), *Hes1* (0.84)/*HES1* (0.88), *Ubc* (0.70)/*UBC* (0.46), *Rhob* (0.75)/*RHOB* (0.26), *Egr1* (1.11)/*EGR1* (1.47), *Nfkbia* (0.91)/*NFKBIA* (0.64), *Klf2* (0.81)/*KLF2* (0.75), *Cdkn1a* (0.94)/*CDKN1A* (0.38), *Fosb* (1.06)/*FOSB* (1.23), *Gimap4* (0.56)/*GIMAP4* (0.76), *Ier5* (0.68)/*IER5* (0.92), *Tsc22d1* (0.53)/*TSC22D1* (0.65), *Tmod3* (0.41)/*TMOD3* (0.78), *Hsp90aa1* (0.61)/*HSP90AA1* (0.87), *Apold1* (0.51)/*APOLD1* (0.53), *Dnajb1* (0.77)/*DNAJB1* (0.60), *Nedd9* (0.35)/*NEDD9* (0.62), *Gimap6* (0.39)/*GIMAP6* (0.47), *Tsc22d3* (0.35)/*TSC22D3* (0.73), *Gas6* (0.45)/*GAS6* (0.60), *Itga6* (0.32)/*ITGA6* (1.29), *Ddx3y* (0.27)/*DDX3Y* (0.93), *Tinagl1* (0.35)/*TINAGL1* (0.39), *Ets1* (0.28)/*ETS1* (0.66), *Timp3* (0.44)/*TIMP3* (0.57), *Slfn5* (0.34)/*SLFN5* (0.75), *Sparcl1* (0.47)/*SPARCL1* (1.04), *Klf4* (0.49)/*KLF4* (1.12), *Sgk1* (0.28)/*SGK1* (0.81) | *Cavin1* (3.95), *Nme2* (2.53), *Cavin3* (2.43), *Selenow* (2.36), *Ccn1* (2.27), *Nop53* (2.21), *Selenop* (2.18), *Atp6v0c* (2.13), *Depp1* (2.13), *Tcim* (2.09)  (889 in total) |
| Human injured vs uninjured |  | *AP000251.3* (3.19), *MTRNR2L12* (3.04), *RP11-777B9.5* (2.96), *MTRNR2L8* (2.93), *MTND6P3* (2.61), *CH507-513H4.5* (2.60), *MSRB3* (2.44), *MTRNR2L10* (2.35), *RP11-475J5.4* (2.32), *AC002075.4* (2.30)  (645 in total) |

**Supplementary Figures**

**Supplementary Figure 1**

**
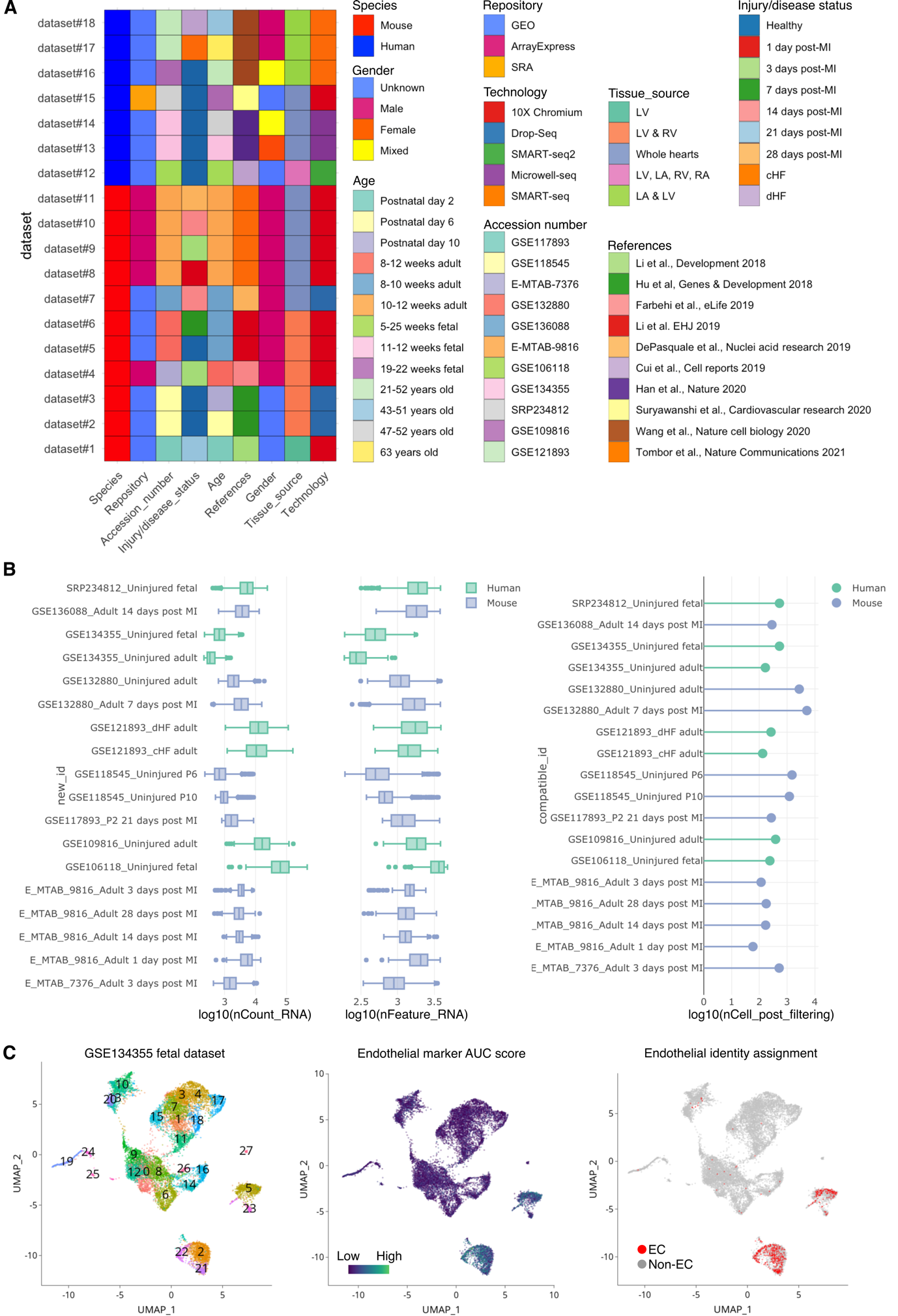
**

**Supplementary Figure 2**


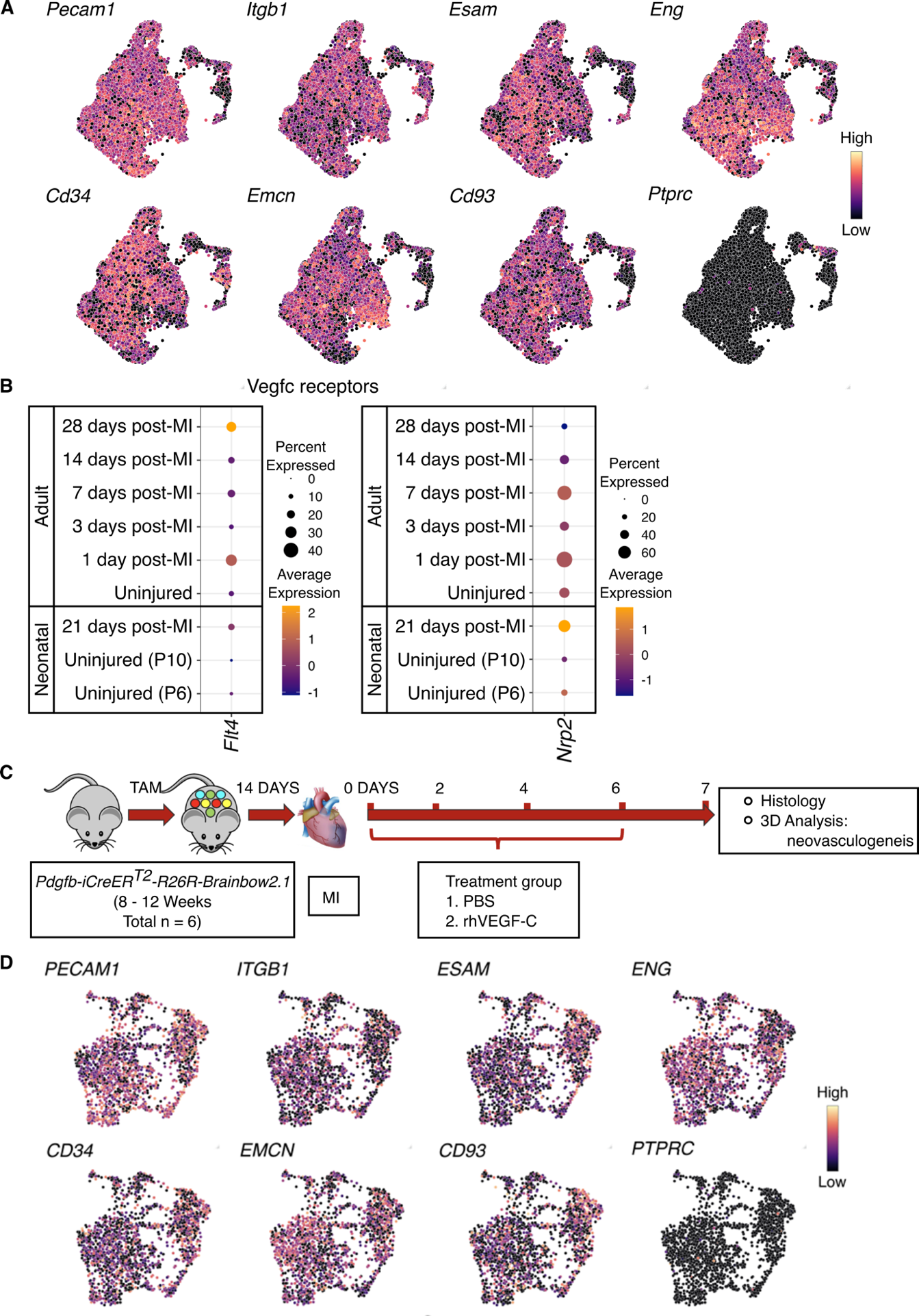


**Supplementary Figure 3**


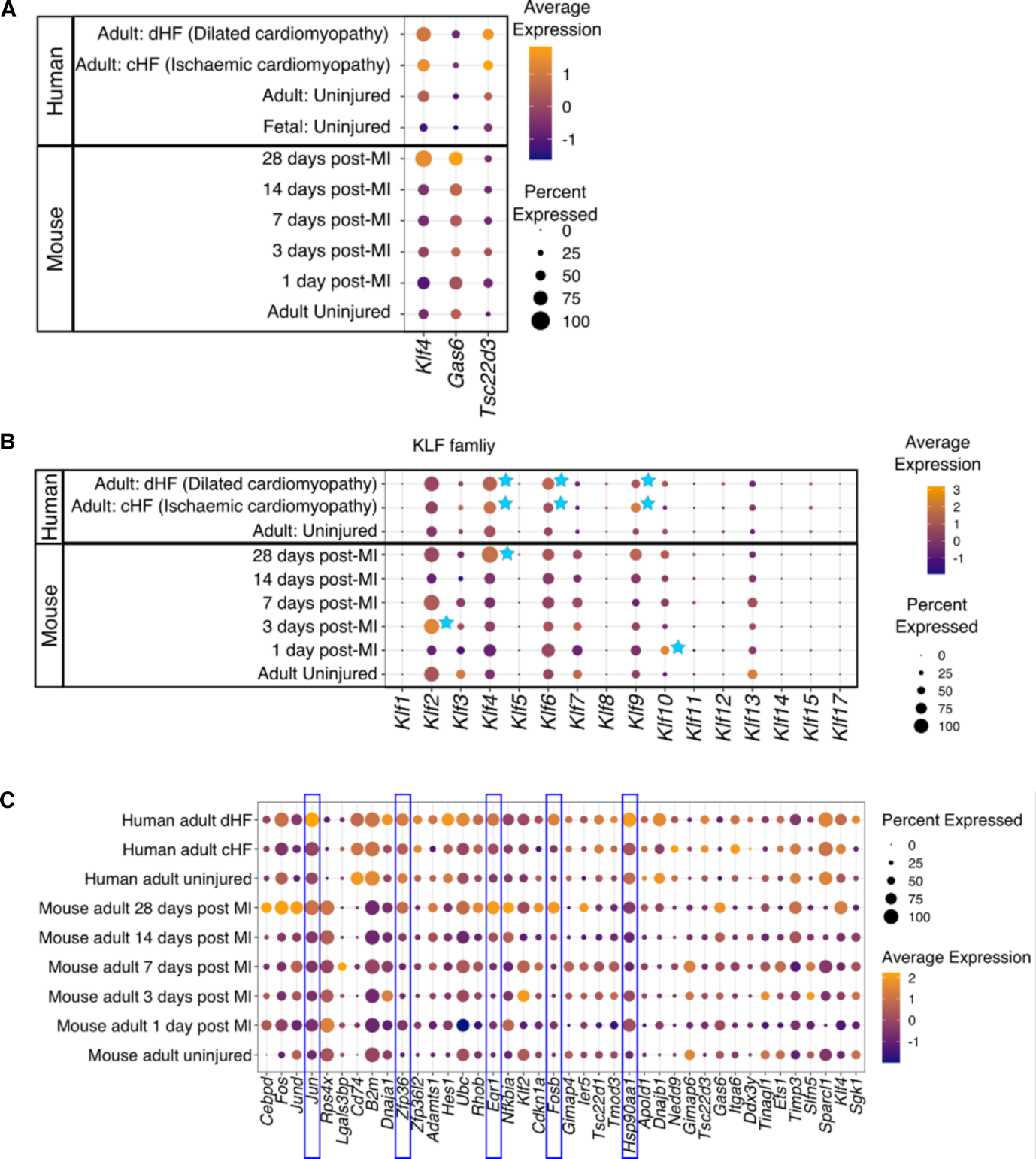


**Supplementary Figure Legends**

**Supplementary Figure 1 Overview of *n* = 18 scRNA-seq datasets used in current study.** **A**. Heatmap indicates the species of origin; the repository where the datasets are deposited; the accession number in corresponding repository; the injury/disease status; the age; the articles where the datasets were published; the gender of the samples; the tissue source; the single cell RNA sequencing technologies used for generating the data. **B**. Boxplots showing properties of each dataset, including the number of counts and the number of features *per* cell and the number of ECs extracted from each dataset (log10 transformed).

**Supplementary Figure 2** **A**. An endothelial identity of the extracted mouse coronary ECs was confirmed by their high expression of markers including *Pecam1*, *Itgb1*, *Esam*, *Eng*, *Cd34*, *Emcn*, and *Cd93* and lack of expression of *Ptprc*. **B**. Dot plots shows the expression level of Vegfc receptors *Flt4* and *Nrp2* in mouse coronary ECs. **C**. Workflow of the *in vivo* study using exogenous rhVEGF-C treatment to augment endogenous neovascularisation *via* endothelial cell clonal proliferation in the adult mouse heart. *Pdgfb-iCreER^T2^-R26R-Brainbow2.1* mice underwent permanent coronary artery ligation and were administered either recombinant human (rh)VEGF-C or PBS control at days 0, 2 4 and 6 post-surgery. Hearts were collected at 7 days for histological profiling and 3D clonal proliferation analysis. **D**. An endothelial identity of the extracted human coronary ECs was confirmed by their high expression of endothelial markers including *PECAM1*, *ITGB1*, *ESAM*, *ENG*, *CD34*, *EMCN*, and *CD93* and rare expression of *PTPRC*.

**Supplementary Figure 3 A**. Expression of *Klf4*, *Gas6* and *Tsc22d3* was upregulated across all time-points in the injured mouse hearts and in both types of human heart failure. cHF, heart failure caused by ischaemic cardiomyopathy; dHF, heart failure caused by dilated cardiomyopathy. **B**. Dot plot shows the expression of Klf family members in human and mouse adult coronary ECs. Blue stars indicate enriched expression in coronary ECs in response to injury e.g., *KLF4*, *KLF6* and *KLF9* in human heart failure and *Klf2*, *Klf4* and *Klf10* in mice post-MI. **C**. Dot plot of 41 genes that were upregulated in both mouse and human cardiac ECs after ischaemic injury, compared to healthy heart coronary ECs. The greatest fold-change in expression in the highest percentage of cells was observed in *Jun*, *Zfp36*, *Egr1*, *Fosb*, and *Hsp90aa1* (blue boxes).
